# Iron controls T helper cell pathogenicity by promoting glucose metabolism in autoimmune myopathy

**DOI:** 10.1002/ctm2.999

**Published:** 2022-08-02

**Authors:** Yimei Lai, Siyuan Zhao, Binfeng Chen, Yuefang Huang, Chaohuan Guo, Mengyuan Li, Baokui Ye, Shuyi Wang, Hui Zhang, Niansheng Yang

**Affiliations:** ^1^ Department of Rheumatology The First Affiliated Hospital Sun Yat‐sen University Guangzhou China; ^2^ Institute of Precision Medicine The First Affiliated Hospital Sun Yat‐sen University Guangzhou China; ^3^ Department of Pediatrics The First Affiliated Hospital Sun Yat‐sen University Guangzhou China

**Keywords:** autoimmune myopathy, glucose metabolism, iron, PFKFB4, T helper cells

## Abstract

**Background:**

T helper cells in patients with autoimmune disease of idiopathic inflammatory myopathies (IIM) are characterized with the proinflammatory phenotypes. The underlying mechanisms remain unknown.

**Methods:**

RNA sequencing was performed for differential expression genes. Gene expression in CD4^+^ T‐cells was confirmed by quantitative real‐time PCR. CD4^+^ T‐cells from IIM patients or healthy controls were evaluated for metabolic activities by Seahorse assay. Glucose uptake, T‐cell proliferation and differentiation were evaluated and measured by flow cytometry. Human CD4^+^ T‐cells treated with iron chelators or *Pfkfb4* siRNA were measured for glucose metabolism, proliferation and differentiation. Signalling pathway activation was evaluated by western blot and flow cytometry. Mouse model of experimental autoimmune myositis (EAM) were induced and treated with iron chelator or rapamycin. CD4^+^ T‐cell differentiation and muscle inflammation in the EAM mice were evaluated.

**Results:**

RNA‐sequencing analysis revealed that iron was involved with glucose metabolism and CD4^+^ T‐cell differentiation. IIM patient‐derived CD4^+^ T‐cells showed enhanced glycolysis and mitochondrial respiration, which was inhibited by iron chelation. CD4^+^ T‐cells from patients with IIM was proinflammatory and iron chelation suppressed the differentiation of interferon gamma (IFNγ)‐ and interleukin (IL)‐17A‐producing CD4^+^ T‐cells, which resulted in an increased percentage of regulatory T (Treg) cells. Mechanistically, iron promoted glucose metabolism by an upregulation of PFKFB4 through AKT‐mTOR signalling pathway. Notably, the knockdown of *Pfkfb4* decreased glucose influx and thus suppressed the differentiation of IFNγ‐ and IL‐17A‐producing CD4^+^ T‐cells. In vivo, iron chelation inhibited mTOR signalling pathway and reduced PFKFB4 expression in CD4^+^ T‐cells, resulting in reduced proinflammatory IFNγ‐ and IL‐17A‐producing CD4^+^ T‐cells and increased Foxp3^+^ Treg cells, leading to ameliorated muscle inflammation.

**Conclusions:**

Iron directs CD4^+^ T‐cells into a proinflammatory phenotype by enhancing glucose metabolism. Therapeutic targeting of iron metabolism should have the potential to normalize glucose metabolism in CD4^+^ T‐cells and reverse their proinflammatory phenotype in IIM.

## Graphical abstract

- Glucose metabolism is enhanced in CD4^+^ T‐cells from IIM patients;
- Iron promotes the differentiation of proinflammatory CD4^+^ T‐cells;
- Iron enhances glucose metabolism in CD4^+^ T‐cells by upregulating PFKFB4 through AKT‐mTOR signaling;
- Iron chelation inhibits autoreactive T‐cell response and prevents autoimmune myositis.

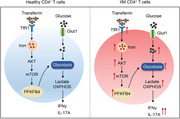

## 1 INTRODUCTION

Dermatomyositis (DM) and polymyositis (PM) are the two most common clinical subsets of idiopathic inflammatory myopathies (IIM) with autoimmune features. DM/PM cause a destruction of muscle cells, leading to muscle weakness. In DM/PM, impaired immunological tolerance is acknowledged by the production of a spectrum of autoantibodies and T‐cell infiltration in the skin and muscle tissues.^1^ The treatment for DM/PM has been advanced during the past decades, but challenges remain for the lack of effective therapeutics.^2^

It has been shown that increased frequency of T helper 17 (Th17) cells was positively correlated with disease severity in DM.^3^ In contrast, the number of FoxP3^+^ regulatory T (Treg) cells was reduced in the skin lesions of DM when compared to that of psoriasis and atopic dermatitis. Compared to healthy controls (HC), the percentage of FoxP3^+^ Treg cells was also reduced in the peripheral blood of DM patients.^4^ In the muscle lesions, CD4^+^ T‐cells, B‐cells and macrophages are the major inflammatory infiltrates in DM, whereas the infiltrates in PM are predominantly CD8^+^ T‐cells and macrophages.^5^ In the skin lesions of DM, it has been shown that majority of infiltrating cells are CD4^+^ T‐cells.^6^ Interferon gamma (IFNγ)‐ and interleukin (IL)‐17A‐producing cells in the lesion of DM/PM promote the expression of toll‐like receptors and inflammatory response in the lesions.^7^, ^8^, ^9^, ^10^ These findings suggest the crucial roles of T helper cells in the pathological inflammation of autoimmune myopathy.

Glucose metabolism is an indispensable biochemical process that provides the very fundamental energy and materials for T‐cell activation, proliferation and differentiation.^11^, ^12^ Glucose metabolism contains two steps that step 1 involves the breakdown of glucose and conversion into pyruvate in the cytoplasm, and step 2 involves the tricarboxylic acid cycle to fuel oxidative phosphorylation (OXPHOS) in mitochondria.^13^ Glucose transporter 1 (Glut1) is responsible for glucose transportation in lymphocytes. Glut1 deficiency decreases the expansion of effector T‐cells and dampens their ability to induce inflammatory disease.^14^ Aerobic glycolysis is preferentially adopted T‐cells when generating proinflammatory helper T‐cells, including IFNγ‐producing Th1 cells and IL‐17A‐producing Th17 cells. Rather than glycolytic metabolism, Treg cells rely on OXPHOS through fatty acid oxidation (FAO) in mitochondria.^15^, ^16^ Metabolic disorders have been noted in T‐cells from autoimmune diseases.^17^, ^18^ The previous study revealed that glycolysis was increased in CD4^+^ T‐cells and inhibition of glucose metabolism ameliorated disease of lupus.^19^ Experimental autoimmune encephalomyelitis (EAE) mice treated with hexokinase (HK) inhibitor 2‐deoxyglucose (2‐DG) showed reduced Th17 cells and increased Treg cells. Clinical outcomes were improved in these mice.^20^ However, the metabolic status of CD4^+^ T‐cells and their contribution to the proinflammatory CD4^+^ T‐cells in IIM are not clear.

Iron metabolism is important for T‐cell activation. In reaction to T‐cell activation, iron demand is rapidly and substantially increased. Iron deficiency caused reduced RORγt and IL‐17A expression in Th17 cells, highlighting the important role of iron in T‐cell differentiation.^21^ In vivo, iron chelation suppressed proinflammatory cytokine expression in T‐cells and ameliorated disease of EAE.^22^ These observations suggest the crucial role of iron homeostasis in autoimmunity. Transferrin receptor 1 (TfR1), encoded by *TFRC*, is important for iron transportation into the cells. Transferrin binds to TfR1 and is internalized by endocytosis to regulate intracellular iron level.^23^ It has been shown that a missense mutation in *TFRC* leads to combined immunodeficiency in human.^24^ The cross‐talk of iron–glucose metabolism influences innate and adaptive immune function. Evidence has indicated that glucose inhibits cellular iron export via elevated expression of hepcidin, whereas iron promotes cellular glucose uptake via OXPHOS.^25^ However, the precise role and physiological significance of iron and the interactions of iron and glucose in T‐cell metabolism and autoimmune myopathy remain elusive.

In this study, our data revealed that CD4^+^ T‐cells from patients with IIM exhibited a higher level of TfR1. IIM CD4^+^ T‐cells showed enhanced glucose metabolic activities and proinflammatory phenotype compared to that of HC. Iron chelation inhibited glucose metabolism and reversed the proinflammatory phenotype of patient's CD4^+^ T‐cells by inhibiting AKT‐mTOR‐PFKFB4 pathway. Iron chelation suppressed autoreactive T‐cell response and prevented autoimmune myopathy in experimental autoimmune myositis (EAM) mice.

## 2 RESULTS

### 2.1 Iron enhances glucose metabolism in CD4^+^ T‐cells from IIM

**FIGURE 1:**
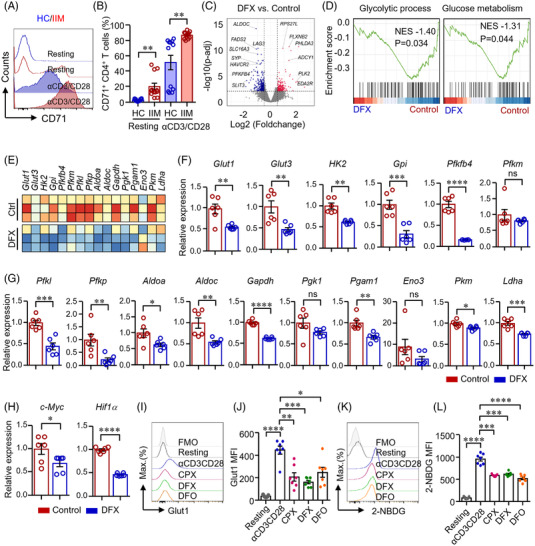
Iron chelation suppressed glucose metabolism in CD4^+^ T‐cells. (A and B) CD4^+^ T‐cells from patients with idiopathic inflammatory myopathies (IIM) or healthy controls (HC) were stimulated with anti‐CD3/CD28 beads for 72 h. Cells were then stained with PE conjugated antibody against CD71 and measured by flow cytometry. Representative histograms were shown. Data from 12 independent samples (dermatomyositis [DM] = 9; polymyositis [PM] = 3). (C)–(E) CD4^+^ T‐cells from HC were stimulated with anti‐CD3/CD28 beads in the presence of absence of deferasirox (DFX, 1 μM) for 72 h. Bulk RNA‐sequencing (RNA‐seq) derived from DFX‐treated and control CD4^+^ cells was performed to screen for differential express genes (DEGs) and enriched gene sets. (C) Volcano plot for DEGs. (D) Gene sets showing the enrichment scores of glycolytic process and glucose metabolism. (E) Heat map of genes for glycolysis derived from bulk RNA‐seq data (*n* = 3). (F)–(H) Gene expression of glucose transporter 1 (*Glut1*), *Glut3*, hexokinase (*Hk*)*2*, *Gpi*, *Pfkfb4*, *Pfkm*, *Pfkl*, *Pfkp*, *Aldoa*, *Aldoc*, *Gapdh*, *Pgk1*, *Pgam1*, *Eno3*, *Pkm*, *Ldha*, *c‐Myc* and *Hif1α* were measured by quantitative real‐time PCR (qPCR). Data were shown as fold change to control group (*n* = 6). (I)–(L) CD4^+^ T‐cells from HC were stimulated with anti‐CD3/CD28 beads for 72 h. Ciclopirox (CPX, 1 μM), DFX (1 μM) and deferoxamine (DFO, 2 μM) were included as indicated. (I and J) CD4^+^ T‐cells were stained with Alexa Fluor 647 conjugated antibody against Glut1 and measured by flow cytometry. Data taken from seven independent samples. (K and L) CD4^+^ T‐cells were incubated with 2‐NBDG (glucose analogue), and glucose uptake was accessed by flow cytometry for the uptake of 2‐NBDG. Mean fluorescence intensity (MFI) is shown. Data from seven independent samples. Data are mean ± SEM. *p < .05, **p < .01, ***p < .001 and ****p < .0001 by Student's *t*‐test in panels (B), (F), (G), (H) and one‐way ANOVA in panels (J and L) followed by adjustments for multiple comparisons. ns, not significant.

Accumulating evidence indicates that TfR1 is important for cellular iron importation. For cellular iron uptake, transferrin binds to TfR1 and is internalized through TfR1‐mediated endocytosis.^26^ Our data showed that TfR1 (CD71) expression was increased in IIM CD4^+^ T‐cells at both resting and activated states as measured by flow cytometry (Figure 1A,B), suggesting that iron might be a participant in IIM CD4^+^ T‐cells. To investigate the potential role of iron in CD4^+^ T‐cells, the cells were treated with iron chelator deferasirox (DFX). We performed bulk RNA‐sequencing (RNA‐seq) to screen for downstream targets of iron. Differentially expressed genes (DEGs) identified by RNA‐seq were shown as a volcano plot that glycolytic‐related genes, including *Aldoc* and *Pfkfb4* and cytokine regulatory gene (*Havcr2*), were downregulated in DFX‐treated CD4^+^ T‐cells. However, p53 target genes, including *Rps27l*, *Phlda3*, *Plk2* and *Eda2r*, were upregulated in DFX‐treated CD4^+^ T‐cells (Figure 1C). For pathway enrichment analysis, the DEGs were assigned into functional pathways of gene ontology (GO), Kyoto Encyclopedia of Genes and Genomes (KEGG), Reactome Pathway Database (RPD) and Wiki Pathways (WP) gene sets. The results showed that iron chelation induced metabolic changes, including glucose metabolism as the modifications in glucose 6−phosphate metabolic process, glycolytic process, glycolytic process through glucose‐6‐phosphate and glycolytic process through the fructose‐6‐phosphate regulation of glycolytic process (Figure S1a). Further analysis by gene set enrichment analysis (GSEA) showed the signalling pathways of glycolytic process and glucose metabolism were significantly downregulated and enriched in DFX‐treated CD4^+^ T‐cells (Figure 1D). Glycolytic genes in CD4^+^ T‐cells were downregulated by iron chelation as by RNA‐seq (Figure 1E), which was further confirmed by quantitative real‐time PCR (qPCR) (Figure 1F,G). The transcripts of *c‐Myc* and *Hif1α*, two key transcription factors in the metabolic pathway,^27^ were also downregulated by iron chelation (Figure 1H). These data imply that iron chelation could lead to a reduction of glucose metabolism in CD4^+^ T‐cells. It is worth noting that iron chelation did not induce cell death (Figure S2), which was consistent with the previous report.^28^

Glut1 is important for glucose transportation in T‐cells.^14^ To further confirm the mRNA expression data, we stimulated CD4^+^ T‐cells with anti‐CD3/CD28 beads for 3 days (d) and found that Glut1 expression in CD4^+^ T‐cells was dramatically increased by anti‐CD3/CD28 stimulation. Notably, Glut1 expression was inhibited by iron chelation (Figure 1I,J). In parallel, glucose uptake by CD4^+^ T‐cells was decreased significantly by iron chelation (Figure 1K,L).

### 2.2 Iron promotes glucose metabolism and differentiation of proinflammatory CD4^+^ T‐cells

**FIGURE 2:**
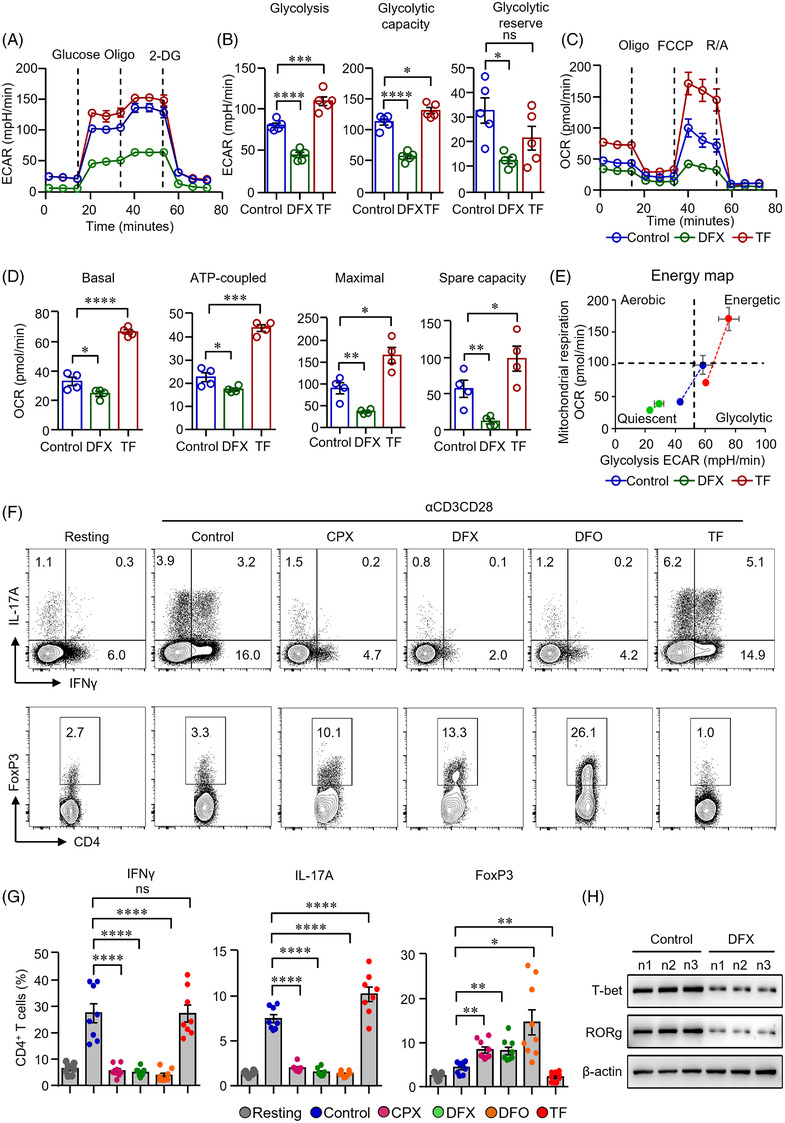
Iron chelation inhibited metabolic activities and modulated the differentiation of CD4^+^ T‐cells. (A)–(E) CD4^+^ T‐cells isolated from healthy controls (HC) were stimulated with anti‐CD3/CD28 beads for 3 d. Ciclopirox (CPX) (1 μM), deferasirox (DFX) (2 μM), deferoxamine (DFO) (2 μM) and transferrin (TF, 50 μg/ml) were included in some of the experiments as indicated. For metabolic activities evaluation of CD4^+^ T‐cells, Glycolysis Stress Test Kit and Mito Stress Test Kit were used to test extracellular acidification rate (ECAR) and oxygen consumption rate (OCR), respectively, by a Seahorse XF96 analyser. (A and B) Glucose, oligomycin (Oligo) and 2‐deoxyglucose (2‐DG) were injected sequentially and ECAR were recorded. ECAR tracing curves and parameters of glycolysis, glycolysis capacity and glycolysis reserve were summarized (*n* = 5). (C and D) Oligo, carbonyl cyanide‐*p*‐trifluoromethoxyphenylhydrazone (FCCP) and rotenone/antimycin A (R/A) were injected sequentially. OCR of basal respiration, respiration coupled to ATP production, maximal respiration and respiratory spare capacity were summarized from four independent samples. (E) Energy map showing metabolic potential of CD4^+^ T‐cells. (F and G) CD4^+^ T‐cells were stimulated with anti‐CD3/CD28 beads in the presence of CPX (1 μM), DFX (2 μM), DFO (2 μM) and TF (50 μg/ml) for 5 d. CD4^+^ T‐cells were stained with Brilliant Violet 421‐conjugated anti‐interferon gamma (IFNγ), APC‐conjugated anti‐interleukin (IL)‐17A and PE‐conjugated anti‐FoxP3 antibodies. Data were acquired by flow cytometry. Representative counter plots were shown and data from eight independent samples. (H) CD4^+^ T‐cells from HC (*n*) were stimulated with anti‐CD3/CD28 beads in the presence or absence of DFX (2 μM) for 5 d. T‐bet and RORg expression was measured by western blot (*n* = 3). All data are mean ± SEM. *p < .05, **p < .01, ***p < .001 and ****p < .0001 by one‐way ANOVA followed by adjustments for multiple comparisons.

After transported into the cells, glucose is converted to pyruvate and then converted to lactate in the cytoplasm, or carbon dioxide and water in the mitochondria, resulting in ATP production to support cellular activities.^13^ To further connect the functions of iron to glucose metabolism, Seahorse extracellular flux assays were applied to measure extracellular acidification rate (ECAR) and oxygen consumption rate (OCR). The Glycolysis Stress Test results revealed that ECAR was decreased when CD4^+^ T‐cells were treated with DFX. Glycolysis, glycolytic capacity and glycolytic reserve were all downregulated by iron chelation in CD4^+^ T‐cells (Figure 2A,B). Moreover, cells were subjected to Mito Stress Test, finding out that basal, ATP‐coupled, maximal respirations OCRs were significantly lower in DFX‐treated CD4^+^ T‐cells (Figure 2C,D). Spare capacity was also decreased by DFX (Figure 2C,D). These data suggest that iron plays a major part in glucose metabolism of CD4^+^ T‐cells. Transferrin is composed of two high‐affinity binding sites for iron Fe^3+^. Transferrin acts as the major transporter for ferric iron through TfR1 and is crucial in the regulation of iron homeostasis.^29^ To further investigate the role of iron in glucose metabolism in CD4^+^ T‐cells, transferrin was added to CD4^+^ T‐cells during activation in parallel. The results revealed that ECAR and OCR were increased dramatically when CD4^+^ T‐cells were fed with transferrin (Figure 2A–D). DFX‐treated CD4^+^ T‐cells were more quiescent at baseline and exhibited lower metabolic potential compared with transferrin‐treated T‐cells (Figure 2E). These data indicate that iron chelation results in decreased glycolysis and mitochondrial respiration whereas iron supplementation promotes them in CD4^+^ T‐cells.

Pathways enrichment analysis derived from RNA‐seq data suggested that iron could be a participant in the activation, differentiation and function of CD4^+^ T‐cells (Figure S1b). TCR signalling pathway was significantly enriched from DEGs between transferrin‐treated and control CD4^+^ T‐cells (Figure S1c). In addition, we found the significant enrichment of pathways regarding the induction or regulation of CD4^+^ T‐cell activation, proliferation, differentiation or homeostasis from DEGs between transferrin‐treated and DFX‐treated CD4^+^ T‐cells (Figure S1d), suggesting that iron would be important in the biological function of CD4^+^ T‐cells.

To further test whether glucose metabolism controlled by iron is functionally linked to CD4^+^ T‐cell differentiation and effector function. Notably, IFNγ and IL‐17A production in CD4^+^ T‐cells was suppressed by all the three iron chelators. Interestingly, the percentages of FoxP3^+^ CD4^+^ T‐cells were increased when iron chelation was performed in CD4^+^ T‐cells (Figure 2F,G). Of note, iron chelation also suppressed of T‐cell growth and proliferation (Figure S3). In contrast, transferrin promoted IFNγ and IL‐17A production but reduced the percentages of FoxP3 in CD4^+^ T‐cells (Figure 2F,G). The replenishment of transferrin reversed the inhibitory effects of iron chelators on CD4^+^ T‐cells. Glucose uptake and cytokine production by CD4^+^ T‐cells were reversed to the control level (Figure S4). To further investigate iron in CD4^+^ T‐cell differentiation, we stimulated CD4^+^ T‐cells with anti‐CD3/CD28 beads. T‐bet and RORg, the key transcription factor for Th1 and Th17 cells, respectively,^30^ were measured by western blot. We found that T‐bet and RORg expression was suppressed by DFX significantly (Figure 2H). Moreover, DFX inhibited IFNγ production under Th1 cell differentiation condition (Figure S5a,b). However, the expression of FoxP3 was not affected under Treg cell differentiation condition (Figure S5c,d), suggesting that iron metabolism was not involved with Treg cell differentiation directly. The change of percentage of FoxP3^+^ Treg cells should be due to the inhibition of proinflammatory T‐cells. Additionally, we found that IFNγ expression in CD8^+^ T‐cells was also downregulated by iron chelation (Figure S6).

### 2.3 Enhanced glucose metabolism in CD4^+^ T‐cells from IIM

**FIGURE 3:**
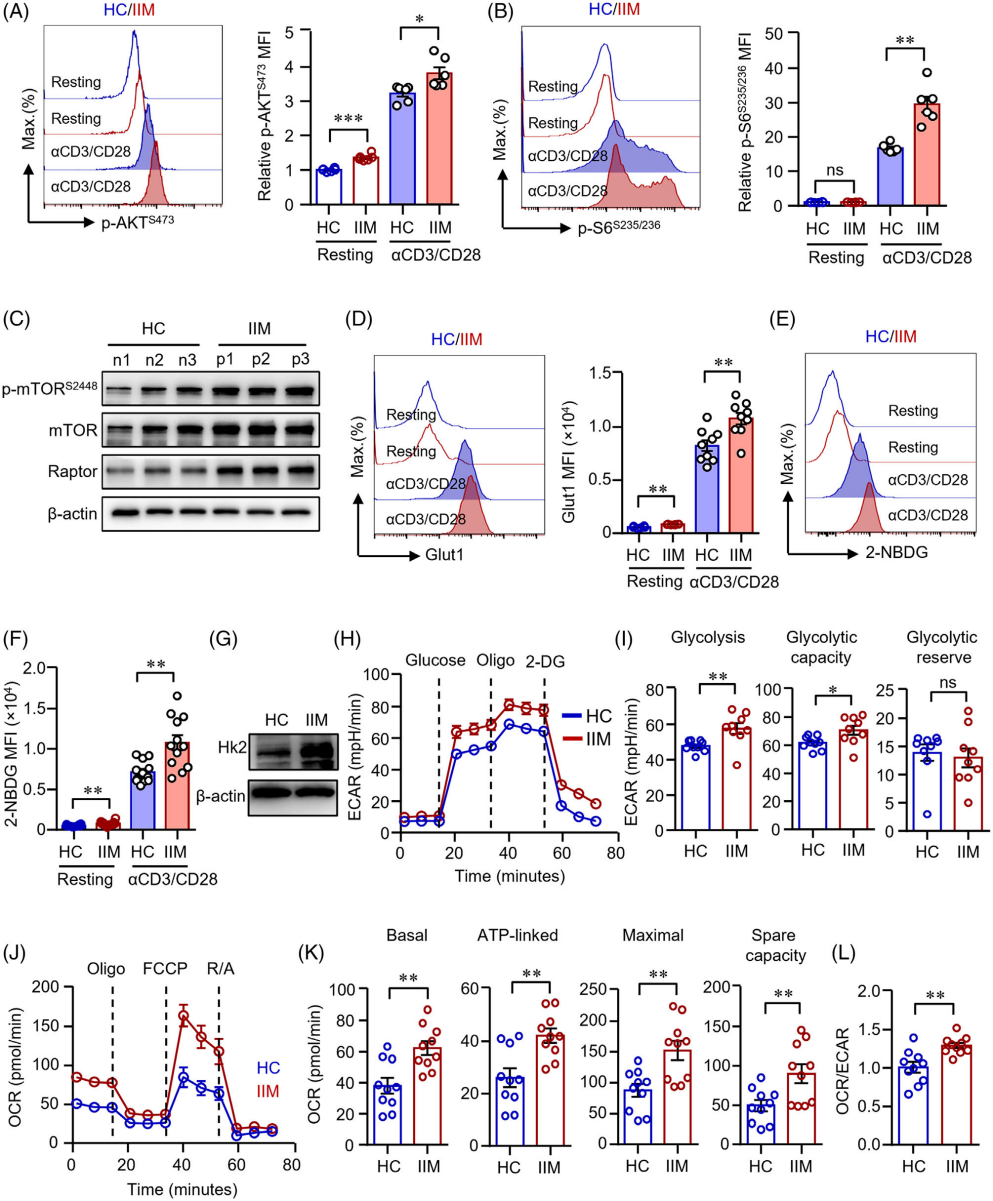
CD4^+^ T‐cells from patients with idiopathic inflammatory myopathies (IIM) showed enhanced glucose metabolism. CD4^+^ T‐cells isolated from patients with IIM or healthy controls (HC) were stimulated with anti‐CD3/CD28 beads for 72 h. (A and B) Phosphorylation of AKT (p‐AKT) and p‐S6 (ribosomal protein) were measured by flow cytometry (dermatomyositis [DM] = 3, polymyositis [PM] = 3). (C) Expression of phosphorylation of mTOR (p‐mTOR), mTOR and Raptor in CD4^+^ T‐cells from IIM patients (p) or HC (*n*) was measured by western blot. Representative bands are shown (*n* = 3). (D) CD4^+^ T‐cells were stained with Alexa Fluor 647‐conjugated antibody against glucose transporter 1 (Glut1) and measured by flow cytometry. Mean fluorescence intensity (MFI) of Glut1 was summarized from nine independent samples (HC = 9, DM = 6, PM = 3). (E and F) CD4^+^ T‐cells were incubated with 2‐NBDG and glucose uptake were evaluated by flow cytometry. MFI of 2‐NBDG was summarized (HC = 12, DM = 8, PM = 4). (G) Hexokinase (Hk)2 expressions in CD4^+^ T‐cells were quantified by western blot. Representative bands of three independent samples. (H)–(L) Glycolytic activities of extracellular acidification rates (ECARs) and mitochondrial activities of oxygen consumption rate (OCR) in CD4^+^ T‐cells were measured using a Seahorse XF96 analyser as in Figure 2. (H and I) ECAR tracing curves and parameters of glycolysis, glycolysis capacity and glycolysis reserve were summarized (HC = 9, DM = 6, PM = 3). (J and K) OCR tracing curves and parameters of mitochondrial function: basal respiration, respiration coupled to ATP production, maximal respiration and respiratory spare capacity were summarized in (K). (L) Ratio of OCR to ECAR. Data from 10 independent samples (HC = 10, DM = 6, PM = 4). All data are mean ± SEM. *p < .05, **p < .01, ***p < .001, ****p < .0001 by Student's *t*‐test. ns, not significant.

Fundamental metabolic processes control immune cell fates.^31^ It has been shown that PI3K‐AKT‐mTOR pathway controls glucose metabolism and aerobic glycolysis, which is essential for T‐cell survival and differentiation.^32^ Elevated mTOR complex 1 (mTORC1) favours the differentiation of CD4^+^ T‐cells into inflammatory Th1 cells, Th2 cells and Th17 lineages.^33^ Our data revealed that the phosphorylation of AKT (p‐AKT) was already increased in IIM CD4^+^ T‐cells at resting state when compared to HC. Consistently, CD4^+^ T‐cells from IIM patients showed an increased expression of p‐AKT at position Ser473 (p‐AKT^S473^) in response to anti‐CD3/CD28 stimulation (Figure 3A). In addition, the phosphorylation of ribosomal protein S6 (p‐S6), downstream of mTOR, was also increased in patients’ CD4^+^ T‐cells as measured by flow cytometry (Figure 3B). Western blot assay further demonstrated the activation of mTORC1 in CD4^+^ T‐cells from IIM patients (Figure 3C). Accordingly, gene expression profile revealed that glycolytic genes required for glucose uptake and breakdown were largely increased in IIM CD4^+^ T‐cells. Gene expressions of *Glut1*, *Hk2*, *Pfk1*, *Pfkfb4*, *Gapdh* and *Ldha* were notably higher in the patient's cells as measured by qPCR. Although *c‐Myc* mRNA was not different between IIM and HC, the transcript of *Hif1α* was increased in IIM CD4^+^ T‐cells (Figure S7).

We next applied flow cytometry to confirm a higher level of Glut1 expression in IIM CD4^+^ T‐cells (Figure 3D). As expected, glucose uptake was higher in IIM CD4^+^ T‐cells (Figure 3E,F). Phosphorylation of glucose mediated by HK is the initial key step in glycolysis pathway. We found that Hk2 expression in IIM CD4^+^ T‐cells was profoundly increased when compared to HC as measured by western blot (Figure 3G).

Glucose metabolism in IIM CD4^+^ T‐cells was further evaluated by Seahorse extracellular flux assays. ECAR and OCR in CD4^+^ T‐cells were analysed 72 h after stimulation with anti‐CD3/CD28 beads. Activated IIM CD4^+^ T‐cells showed significantly higher glycolysis and glycolytic capacity when compared to control CD4^+^ cells (Figure 3H,I). In parallel, patient‐derived CD4^+^ T‐cells had increased basal, ATP‐linked, maximal respiration, as well as higher level of mitochondrial reservation (Figure 3J,K). Patient's CD4^+^ T‐cells were able to use oxygen more effectively than control cells. Although ECAR and OCR were both elevated in CD4^+^ T‐cells from patients, the OCR/ECAR ratio was increased in patient's cells (Figure 3L), suggesting higher mitochondrial respiratory reservation and better preparation for a higher level of glucose metabolism in patient's CD4^+^ T‐cells.

### 2.4 Iron promotes glucose metabolism and proinflammatory phenotype of CD4^+^ T‐cells in IIM

**FIGURE 4:**
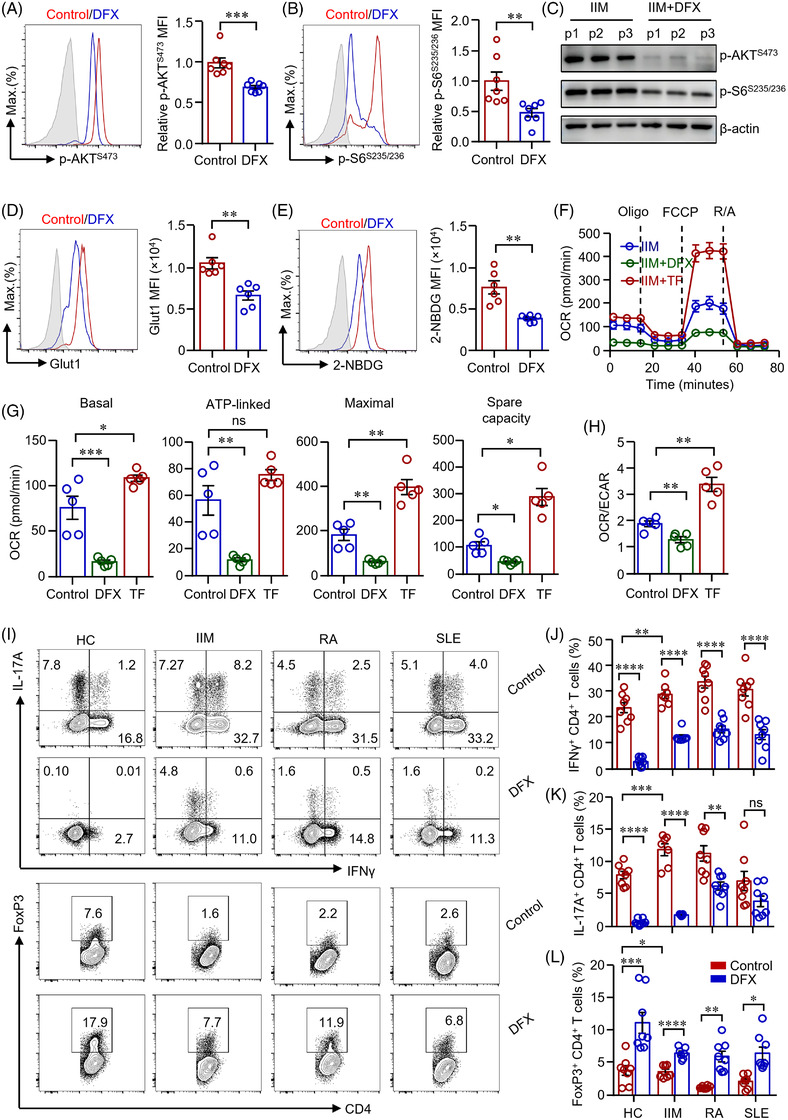
Iron chelation suppressed glucose metabolism and inhibited effector function of CD4^+^ T‐cells from patients with idiopathic inflammatory myopathies (IIM). (A)–(H) CD4^+^ T‐cells from patients with IIM were stimulated with anti‐CD3/CD28 beads for 72 h. Deferasirox (DFX) (1 μM) or transferrin (TF) (50 μg/ml) was included as indicated. (A and B) Cells were stained with antibodies against phosphorylation of AKT (p‐AKT) and p‐S6 (ribosomal protein), respectively. Cells were then stained with Alexa Fluor 594‐conjugated goat anti‐rabbit IgG and measured by flow cytometry (dermatomyositis [DM] = 4, polymyositis [PM] = 3). p‐AKT and p‐S6 in CD4^+^ T‐cells from patients (p) with IIM were further measured by western blot (C, *n* = 3). (D) Cells were stained with Alexa Fluor 647‐conjugated antibody against glucose transporter 1 (Glut1) and measured by flow cytometry. Mean fluorescence intensity (MFI) of Glut1 was summarized (DM = 3, PM = 3). (E) Representative histograms of 2‐NBDG uptake by CD4^+^ T‐cells as measured by flow cytometry and MFI were summarized (DM = 3, PM = 3). (F)–(H) Extracellular acidification rate (ECAR) and oxygen consumption rate (OCR) of CD4^+^ T‐cells were measured by a Seahorse XF96 analyser as in Figure 2. (F and G) OCR of basal respiration, respiration coupled to ATP production, maximal respiration and respiratory spare capacity were summarized (DM = 3, PM = 2). (H) Ratio of OCR to ECAR. (I)–(L) CD4^+^ T‐cells from patients with IIM, rheumatoid arthritis (RA) and systemic lupus erythematosus (SLE) were stimulated with anti‐CD3/CD28 beads in the presence or absence of DFX (1 μM) for 5 d. Representative counter plots for interferon gamma (IFNγ), interleukin (IL)‐17A and FoxP3 expression in CD4^+^ T‐cells as measured by flow cytometry (DM = 4, PM = 3, healthy controls [HC], RA and SLE = 8). All data are mean ± SEM. *p < .05, **p < .01, ***p < .001 and ****p < .0001 by Student's *t*‐test in panels (A), (B), (D), (E), (J)–(L) and one‐way ANOVA in panels (G and H) followed by adjustments for multiple comparisons.

The previous data showed increased glucose metabolism in IIM CD4^+^ T‐cells. We were to test whether iron chelation could suppress glucose metabolism and reversed the proinflammatory phenotype of IIM CD4^+^ T‐cells. p‐AKT and S6 in IIM CD4^+^ T‐cells was measured by western blot and flow cytometry, respectively. Notably, iron chelator DFX effectively decreased the expression of p‐AKT^S473^ and p‐S6^S235/236^ in IIM CD4^+^ T‐cells as measured by flow cytometry and western blot, respectively (Figure 4A–C). The inhibition of AKT‐mTOR signalling resulted in decreased Glut1 expression in CD4^+^ T‐cells (Figure 4D). Glucose uptakes by IIM CD4^+^ T‐cells were also suppressed by DFX effectively (Figure 4E). To further study the role of iron in glucose metabolism of CD4^+^ T‐cells from IIM, cells were subjected to Seahorse extracellular flux assays. We found that iron chelation reduced oxygen consumption by patient's CD4^+^ T‐cells significantly. Moreover, transferrin increased oxygen consumption by patient's CD4^+^ T‐cells notably (Figure 4F,G). The ratio of OCR/ECAR was reduced by DFX but increased by transferrin (Figure 4H). These data show the critical role of iron in glucose metabolism in CD4^+^ T‐cells in from IIM patients.

The differentiation of Th1 and Th17 cells relies on glucose glycolysis, whereas the differentiation of Treg cells relies on FAO.^15^ To find out how iron regulates CD4^+^ T‐cell differentiation in IIM through regulating glucose metabolism, IIM CD4^+^ T‐cells were treated with DFX during the activation by anti‐CD3/CD28 beads. CD4^+^ T‐cells from patients with rheumatoid arthritis (RA) and systemic lupus erythematosus (SLE) were used as disease controls. We found that the production of IFNγ and IL‐17A was increased in IIM CD4^+^ T‐cells (Figure 4I–K), which was in‐line with the increased glucose metabolism in patient T‐cells (Figure 3). Iron chelation induced to reduce IFNγ and IL‐17A production in IIM CD4^+^ T‐cells dramatically (Figure 4I–K). Our further result showed that the frequency of Treg cells was decreased in patients with IIM, which was restored by DFX treatment (Figure 4I,L). Similar results were found in CD4^+^ T‐cells from RA and SLE, except that IL‐17A production was not affected by DFX in CD4^+^ T‐cells from RA (Figure 4I–L). Although DFX reduced CD71 expression in CD4^+^ T‐cells from patients with IIM, RA, SLE or HC, CD71 expression in CD4^+^ T‐cells did not differ between RA, SLE and HC (Figure S8). These data indicate that iron plays a broad and essential role in modulating CD4^+^ T‐cell differentiation in general. However, iron may not be the major contributor to CD4^+^ T‐cell activation in patients with RA or SLE, pointing to a role of iron specific for CD4^+^ T‐cells from IIM.

### 2.5 Iron controls glucose metabolism in CD4^+^ T‐cells through PFKFB4

**FIGURE 5:**
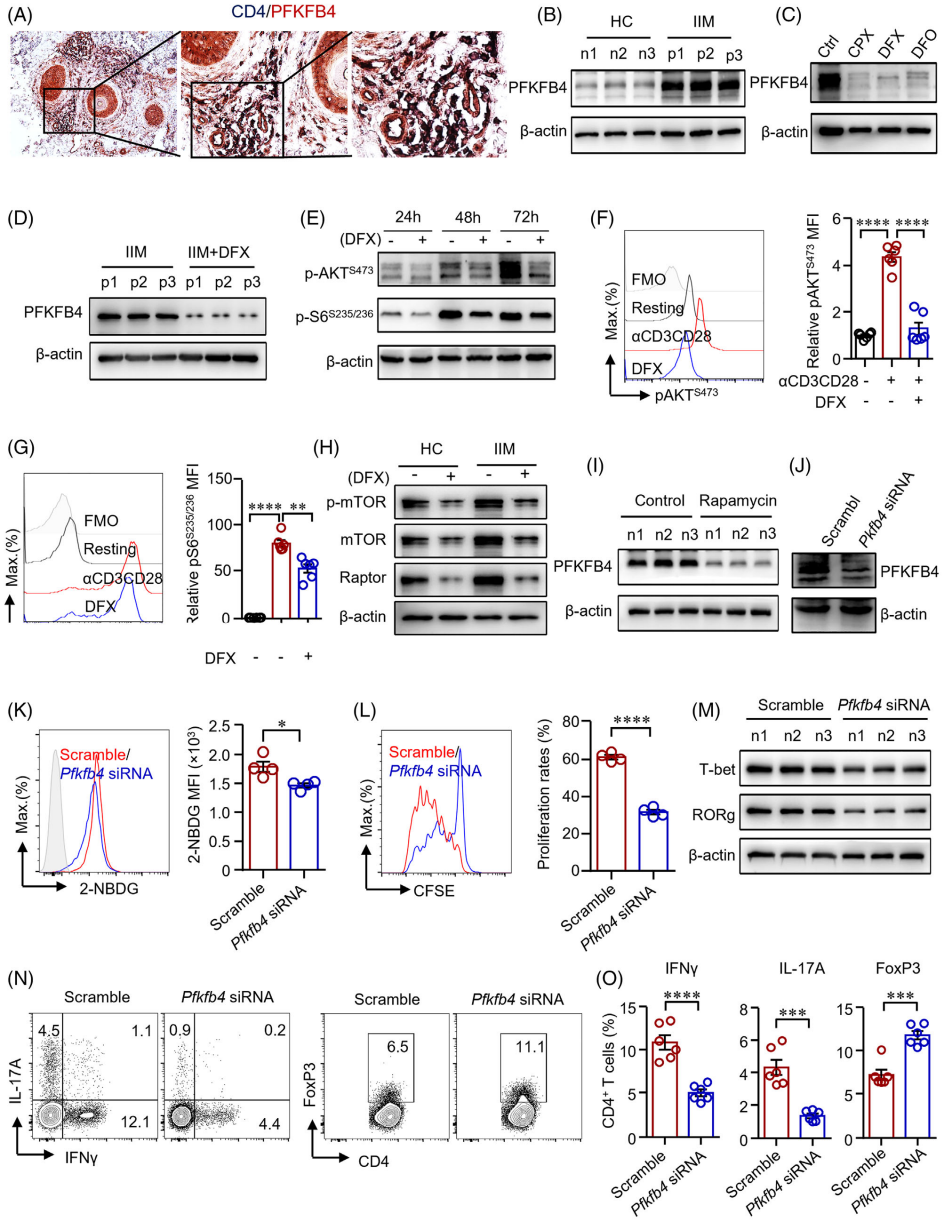
Iron promotes glucose metabolism in CD4^+^ T‐cells through PFKFB4 in idiopathic inflammatory myopathies (IIM). (A) Double staining of CD4 (dark blue) and PFKFB4 (red) of skin sections from patients with IIM by immunohistochemistry. Original magnification: 200×. (B)–(I) CD4^+^ T‐cells were stimulated with anti‐CD3/CD28 beads for 72 h in the presence of ciclopirox (CPX) (1 μM), deferasirox (DFX) (2 μM), deferoxamine (DFO) (2 μM) or rapamycin (10 nM) as indicated. PFKFB4 expression in CD4^+^ T‐cells from IIM patients (p) or healthy controls (HC) (*n*) (B), or CD4^+^ T‐cells from HC treated with iron chelators (C), or CD4^+^ T‐cells from IIM treated with DFX (D) was measured by western blot. Representative bands of three independent samples. (E) Phosphorylation of AKT (p‐AKT) and p‐S6 (ribosomal protein) expression in CD4^+^ T‐cells from HC was measured by western blot. (F and G) CD4^+^ T‐cells from HC were stained with primary antibodies against p‐AKT and p‐S6, followed by Alexa Fluor 594‐conjugated goat anti‐rabbit IgG and measured by flow cytometry (*n* = 6). (H) Expression of phosphorylation of mTOR (p‐mTOR), mTOR and Raptor in CD4^+^ T‐cells from IIM or HC treated with or without DFX was measured by western blot. Representative bands of three independent samples. (I) PFKFB4 expression in CD4^+^ T‐cells from HC treated with rapamycin was measured by western blot (*n* = 3). (J)–(O) CD4^+^ T‐cells from HC or IIM were electrotransfected with *Pfkfb4*‐ or scramble siRNA. (J) *Pfkfb4* knockdown efficiency was confirmed by western blot. (K) Uptake of 2‐NBDG by HC CD4^+^ T‐cells was measured by flow cytometry (*n* = 4). (L) CD4^+^ T‐cells from HC were labelled with carboxyfluorescein succinimidyl ester (CFSE) and cell proliferation was calculated for the dilution of CFSE as measured by flow cytometry (*n* = 4). (M) T‐bet and RORg expression in CD4^+^ T‐cells from HC was measured by western blot (*n* = 3). (N and O) Interferon gamma (IFNγ), interleukin (IL)‐17A and FoxP3 expression in CD4^+^ T‐cells from HC measured by flow cytometry. Data are mean ± SEM. **p < .01, ***p < .001 and ****p < .0001 by one‐way ANOVA followed by adjustments for multiple comparisons in panels (F and G) and Student's *t*‐test in panels (K), (L) and (O).

The RNA‐seq data revealed that *Pfkfb4* was among the predominant changed genes in DFX‐treated CD4^+^ T‐cells as well as in IIM patient‐derived CD4^+^ T‐cells (Figures 1 and S7). Immunohistochemistry staining showed that PFKFB4 was highly expressed in infiltrated CD4^+^ T‐cells in the skin lesions of patients with IIM (Figure 5A). PFKFB4 expression in IIM CD4^+^ T‐cells was further measured by western blot. The data confirmed that PFKFB4 expression was dramatically increased in patient's CD4^+^ T‐cells (Figure 5B). To link iron metabolism to PFKFB4 expression, CD4^+^ T‐cells from HC were treated with iron chelators of ciclopirox (CPX) olamine, DFX and deferoxamine (DFO) mesylate salt. The result showed that iron chelation suppressed the expression of PFKFB4 in CD4^+^ T‐cells dramatically (Figure 5C). We further confirmed that iron chelation decreased PFKFB4 expression in CD4^+^ T‐cells from IIM patients (Figure 5D). Pathway enrichment derived from DEGs of the RNA‐seq data as shown in Figure S1 suggested that PI3K‐AKT‐mTOR pathway was involved in the DFX‐induced metabolic changes. In addition, enrichment results of DEGs from comparison of transferrin versus control and DFX versus transferrin categories demonstrated the involvement of PI3K‐AKT‐mTOR pathway in the downstream of iron homeostasis. We thus measured the p‐AKT and S6 in CD4^+^ T‐cells treated with iron chelation by western blot and flow cytometry, respectively, finding out that the expressions of p‐AKT^S473^ and p‐S6^S235/236^ decreased notably by iron chelation (Figure 5E–G), suggesting iron controls PFKFB4 expression through AKT‐mTOR signalling. To confirm this hypothesis, mTORC1 expression in DFX treated‐CD4^+^ T‐cells from HC or IIM was measured by western blot, finding out that iron chelation suppressed mTORC1 notably (Figure 5H). Further, PFKFB4 expression was reduced by mTOR inhibitor rapamycin as measured by western blot (Figure 5I). Together, iron controls PFKFB4 expression through AKT‐mTOR pathway.

To further uncover the role of PFKFP4 in CD4^+^ T‐cell differentiation, *Pfkfb4* was knocked down by *Pfkfb4* siRNA (Figure 5J). We found that knockdown of *Pfkfb4* led to decreased glucose uptake by CD4^+^ T‐cells (Figure 5K). T‐cell proliferation was suppressed when *Pfkfb4* was knocked down (Figure 5L). Notably, the knockdown of *Pfkfb4* suppressed the expression of T‐bet and RORg in CD4^+^ T‐cells (Figure 5M), which indicated a role of PFKFB4 in the differentiation of Th1 and Th17 cells. Knockdown of *Pfkfb4* reduced IFNγ production in CD4^+^ T‐cells significantly. In addition, the IL‐17A^+^CD4^+^ T‐cells were substantially decreased by iron chelation (Figure 5N,O). In contrast, the knockdown of *Pfkfb4* induced the percentage of FoxP3^+^ CD4^+^ T‐cells (Figure 5N,O), which was further confirmed using CD4^+^ T‐cells from patients with IIM (Figure S9). Consistently, the knockdown of *Pfkfb4* in naïve CD4^+^ T‐cells suppressed the production of IFNγ production under Th1 cell differentiation condition (Figure S10).

### 2.6 Iron promotes autoreactive T‐cells during autoimmune myositis

**FIGURE 6:**
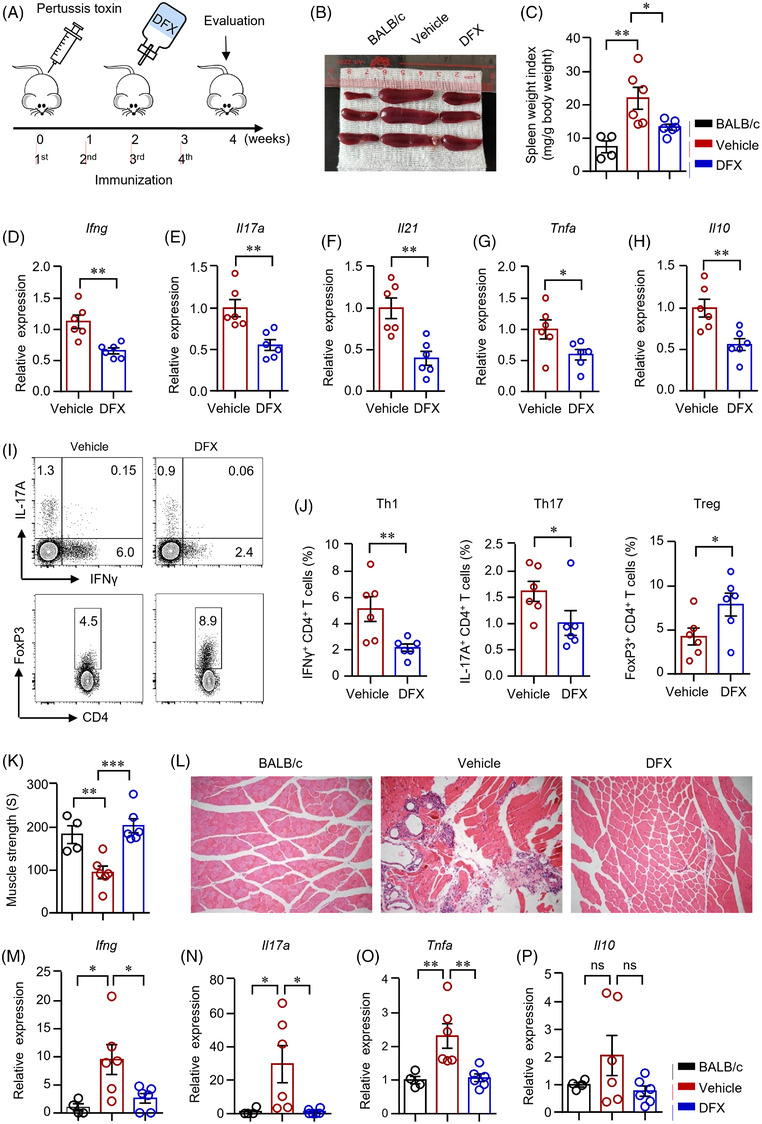
Iron chelation suppressed CD4^+^ T‐cell response during autoimmune myositis. (A) Scheme of the mouse experiment. Experimental autoimmune myositis (EAM) was induced as described in Section 4. Mice were first injected with 500‐ng pertussis toxin intraperitoneally and then immunized with myosin (1 mg) emulsified with complete Freund's adjuvant four times at 1‐week interval. The EAM mice were fed with deferasirox (DFX) (30 mg/kg/d) dissolved in the drinking water after the second immunization for 2 weeks. (B and C) Spleen weight index as calculated spleen weight (mg) to body weight (g) 4 weeks after the primary immunization. (D)–(H) *Ifng, Il17a*, *IL21*, *Tnfa* and *Il10* gene expression in the draining lymph nodes were measured by quantitative real‐time PCR (qPCR). (I and J) Interferon gamma (IFNγ)‐, interleukin (IL)‐17A‐ and FoxP3‐producing CD4^+^ T‐cells in draining lymph nodes were measured by flow cytometry. Representative counter plots were shown. (K) Muscle strength of the EAM mice treated with DFX or vehicle. (L) Muscle (quadriceps) sections of mice treated with DFX or vehicle were stained with hematoxylin and eosin. Representative images are shown. Original magnification: 100×. (M)–(P) RNA was extracted from muscle tissue of quadriceps. Transcripts of *Ifng*, *Il17a*, *Tnfa* and *Il10* in the muscle tissues were quantified by qPCR. All data are mean ± SEM. *n* = 6. *p < .05, **p < .01, by one‐way ANOVA followed by adjustments for multiple comparisons in panels (C), (K), (M)–(P) and Student's *t*‐test in panels (D)–(H) and (J). ns, not significant.

To investigate the role of iron in autoimmune myopathy, an EAM mouse model was established by immunizing the mice with autologous myosin (Figure 6A). Experimental mice were treated with DFX or vehicle for 2 weeks. We found that spleen size was increased dramatically after immunization and iron chelation by DFX decreased the size of spleen significantly (Figure 6B,C). The expression of *Ifng*, *Il17*a, *Il21*, *Tnfa* and *Il10* in cells from the draining lymph nodes was determined by qPCR, which revealed that iron chelation decreased gene expressions of *Ifng*, *Il17*a, *Il21*, *Tnfa* and *Il10* notably (Figure 6D–H). To further test cytokine production by CD4^+^ T‐cells from DFX or vehicle treated mice, cells were isolated from draining lymph nodes and cytokine production in CD4^+^ T‐cells was measured. The data showed that mice treated with DFX had significantly lower percentages of IFNγ^+^‐ and IL‐17A^+^ CD4^+^ T‐cells (Figure 6I,J). Further, EAM mice treated with DFX showed lower percentages of double (IFNγ^+^IL‐17A^+^, IFNγ^+^TNFα^+^, IL‐17A^+^TNFα^+^) and triple positive (IFNγ^+^IL‐17A^+^TNFα^+^) CD4^+^ T‐cells, suggesting that iron chelation by DFX suppressed the proinflammatory multifunctional CD4^+^ T‐cells in EAM effectively (Figure S11). As expected, cytokine production by CD8^+^ T‐cells were also profoundly reduced by DFX in EAM (Figure S12). In consistent with the in vitro data, mice treated with DFX showed a higher percentage of FoxP3^+^CD4^+^ T‐cells in the draining lymph nodes (Figure 6I,J).

IIM is characterized by diffuse muscle weakness and T‐cell infiltration in skin and muscle tissues pathologically.^34^ Immunization led to decreased muscle strength in the EAM mice. Pathologic analysis further demonstrated immune infiltration to the muscle tissue of EAM mice, which confirmed the establishment of myopathies in the mice (Figure 6L). Iron chelation enhanced muscle strength (Figure 6K). DFX treatment also decreased immune infiltration into the muscle tissue (Figure 6L). Importantly, gene expressions of *Ifng*, *Il17*a, *Il21*, *Tnfa* in muscle tissues of quadriceps were decreased effectively by DFX treatment. Levels of *Il10* were largely unaffected in DFX‐treated mice (Figure 6M–P). Collectively, iron chelation suppressed Th1 and Th17 cell differentiation but increased Treg differentiation during autoimmune myositis, which led to decreased muscle inflammation and increased muscle strength.

### 2.7 Rapamycin inhibits CD4^+^ T‐cell response in autoimmune myositis by suppressing PFKFB4

**FIGURE 7:**
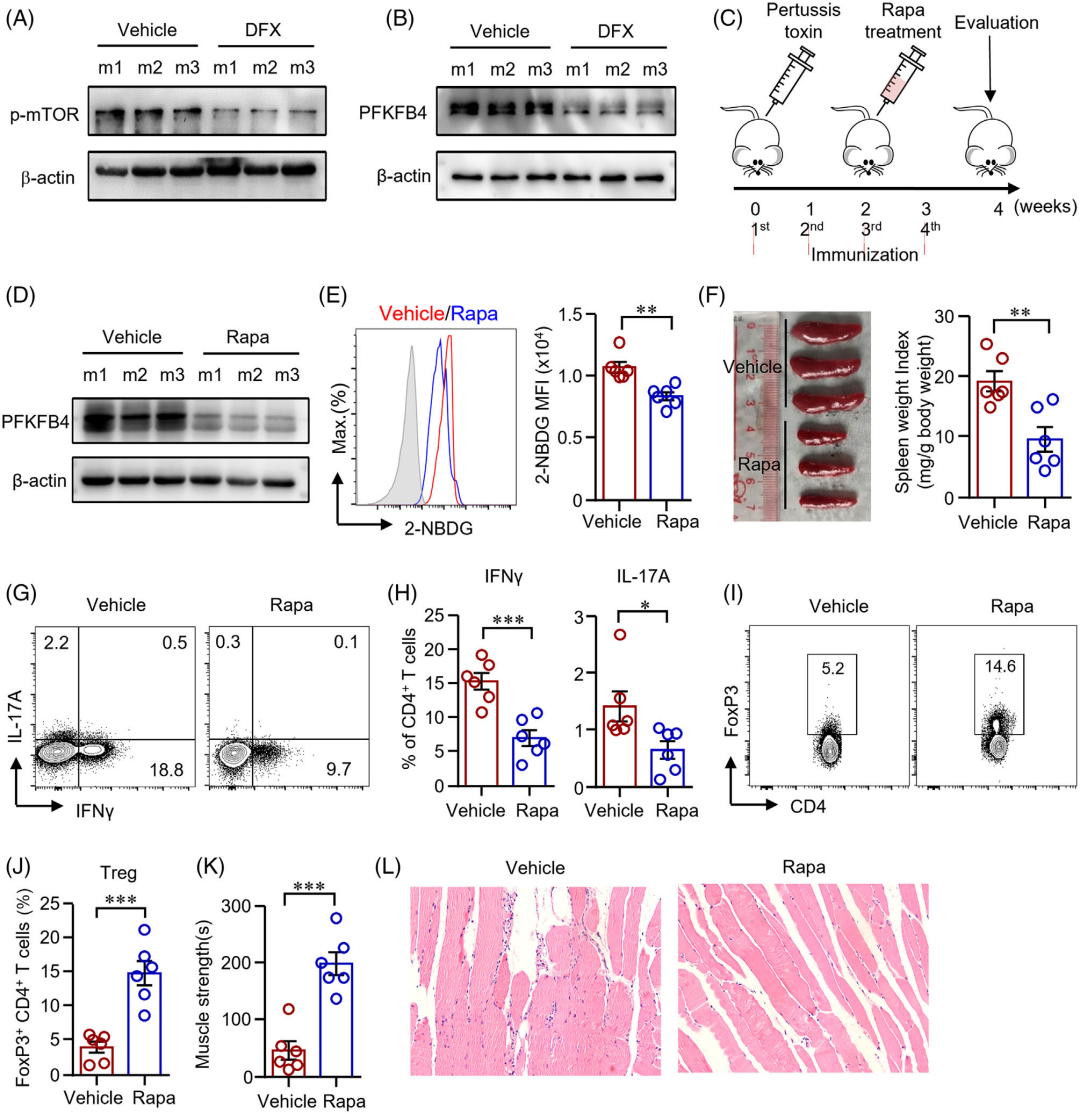
Rapamycin suppressed PFKFB4 and reduced CD4^+^ T‐cell response during autoimmune myositis. (A and B) CD4^+^ T‐cells from mice (m) treated with deferasirox (DFX) or vehicle were stimulated with anti‐CD3/CD28 antibodies. Phosphorylation of mTOR (p‐mTOR) and PFKFB4 expression in CD4^+^ T‐cells were measured by western blot. Representative bands were shown. (C) Scheme of the mouse experiment. Experimental autoimmune myositis (EAM) was induced as described in Figure 6. The EAM mice were treated with rapamycin (Rapa, 1.5 mg/kg/d) or vehicle after the second immunization for 2 weeks. (D) PFKFB4 expression in CD4^+^ T‐cells from mice treated with Rapa or vehicle was measured by western blot. (E) Uptake of 2‐NBDG by CD4^+^ T‐cells from mice treated with Rapa or vehicle was measured by flow cytometry. (F) Spleen weight index as calculated spleen weight (mg) to body weight (g). (G)–(J) Interferon gamma (IFNγ), interleukin (IL)‐17A and FoxP3 expression in CD4^+^ T‐cells from draining lymph nodes were measured by flow cytometry. Representative counter plots are shown. (K) Muscle strength of the EAM mice treated with rapamycin or vehicle. (L) Representative images of hematoxylin and eosin stain of muscle (quadriceps) sections from mice treated with Rapa or vehicle. Original magnification: 200×. All data are mean ± SEM. *n* = 6. *p < .05, **p < .01, ***p < .001 by Student's *t*‐test.

To further uncover the mechanisms of iron in regulating CD4^+^ T‐cells in EAM, we isolated cells from mice treated with DFX or vehicle as in Figure 6. CD4^+^ T‐cells from DFX‐treated EAM mice showed reduced mTOR signalling. Phosphorylated mTOR was decreased by iron chelation as measured by western blot (Figure 7A). Further, PFKFB4 expression in CD4^+^ T‐cells from DFX‐treated EAM mice was downregulated by iron chelation (Figure 7B). These data were consistent with the in vitro data that iron controlled CD4^+^ T‐cells through mTOR signalling. Data have shown the effects of mTOR inhibition in the treatment of EAM.^35^ Here, we further investigated the underlying mechanisms. EAM mice were induced as in Figure 6. EAM mice were treated with mTOR‐specific inhibitor rapamycin or vehicle (Figure 7C). Western blot analysis revealed that PFKFB4 expression in CD4^+^ T‐cells from rapamycin‐treated EAM mice was dramatically reduced (Figure 7D). Further, CD4^+^ T‐cells from rapamycin‐treated EAM mice showed decreased glucose uptake as measured by flow cytometry (Figure 7E), which is in‐line with the in vitro data that mTOR controlled glucose metabolism in CD4^+^ T‐cells through PFKFB4. Size of spleen was significantly smaller in EAM mice treated with rapamycin (Figure 7F). Rapamycin treatment decreased Th1/Th17 cells and increased Treg cells (Figure 7H–J), resulting in stronger muscle strength and ameliorated muscle inflammation in the EAM mice (Figure 7K,L).

## 3 DISCUSSION

The activation of adaptive immunity is critical for the development and progression of IIM. In this study, we identified a hallmark of IIM T‐cells that iron enhanced glucose metabolism in patient's CD4^+^ T‐cells. Reprogramed bioenergetic strategies of CD4^+^ T‐cells by iron promoted their proinflammatory functions in IIM. Mechanistically, we showed that iron enhanced glucose metabolism and promoted the proinflammatory IFNγ‐ and IL‐17A–producing T‐cells through AKT‐mTOR‐PFKFB4 pathway. Iron chelation suppressed autoreactive T‐cells and prevented autoimmune myositis. Collectively, iron functions as a metabolic regulator of CD4^+^ T‐cells and turns them into proinflammatory effector T‐cells in IIM.

Upon infiltration into the skin or muscle tissues, autoreactive T‐cells undergo the activation and production of proinflammatory cytokines.^34^, ^36^ The previous study revealed that local iron accumulation in the central nervous system could be a critical mechanism to promote cytokine production during T‐cell reactivation.^22^ However, the specific function of iron in autoimmune myopathy is not known. Our data revealed the increased expression TfR1 in IIM CD4^+^ T‐cells, suggesting that iron might be involved in the pathogenesis of IIM by regulating CD4^+^ T‐cells. Iron is an essential cofactor in a broad range of cellular processes.^23^ In addition to its role in oxygen uptake in red blood cells, iron is required for mitochondria to maintain its functions, biosynthesis, electron transport and OXPHOS.^37^ Iron supply maintained mitochondrial respiration.^38^ In contrast, mitochondrial respiration was suppressed in iron‐deficient cardiomyocytes.^39^ Here, we showed that iron chelation suppressed glycolysis and mitochondrial respiration in CD4^+^ T‐cells from HC as well as patients with IIM. The suppressed metabolism by iron chelation would lead to dampened proinflammatory T‐cell differentiation. Our study thus provides new therapeutic opportunities for the treatment of IIM.

Interruption of cellular intrinsic metabolic pathways has been shown to regulate immunity. As a result, metabolic dysregulation can serve as a risk factor for cancer and immunological diseases.^18^, ^40^ Dysregulated mitochondrial function and fatty acid metabolism promoted tissue invasiveness of T‐cells in RA,^41^, ^42^ whereas CD4^+^ T‐cells from lupus patients were predominantly enhanced with glucose glycolysis and mitochondrial respiration.^19^ Inhibition of glutaminase 1 was effective in attenuating lupus‐like disease in MRL/lpr mice and EAE by reducing Th17 cells.^43^ In the Guillain–Barré syndrome, the inhibition of glycolysis decreased the numbers of Th1/Th17 cells and upregulating Treg‐cell population, resulting in milder experimental autoimmune neuritis.^44^ Thus, targeting metabolic pathways and reprogramming T‐cell metabolism are promising strategies to treat autoimmune diseases.^45^ Our data emphasize the contribution of metabolism dysregulation in CD4^+^ T‐cells during autoimmune myositis. Enhanced metabolic activities of CD4^+^ T‐cells render their proinflammatory potency in IIM.

In T‐cells, mTORC1 is important for cellular aerobic glycolysis, which posts great impacts on T‐cell differentiation and effector function.^33^ mTORC1 activated by the PI3K/AKT pathway has been the central in reprogramming gene expression in support of glucose glycolysis. mTORC1 signalling favours the Th1 and Th17 cell differentiations by promoting glycolysis. However, mTORC1‐mediated glucose glycolysis restricts the Treg‐cell differentiation.^12^, ^46^ Here we observed that CD4^+^ T‐cells from patients with IIM exhibited upregulated mTORC1 signalling, together with enhanced glucose metabolism. Notably, iron chelation suppressed AKT‐mTOR signalling. The previous report showed that iron chelation inhibits mTORC1 signalling in cancer cells.^47^ In‐line with these reports, our data showed that mTORC1 was inhibited in CD4^+^ T‐cells from patients with IIM or HC when iron was chelated. Considering the importance of mTORC1 for CD4^+^ T‐cell activation and differentiation, iron represents a pivotal role for the fate of CD4^+^ T‐cells.

Evidence has shown that Th1‐ and Th17‐cell differentiation is suppressed, whereas Treg‐cell differentiation is increased under glucose‐restricted conditions.^15^ Here, by enhancing glucose glycolysis in CD4^+^ T‐cells, iron promoted Th1 and Th17 cells. Moreover, EAM mice treated with iron chelator showed reduced percentages of Th1 and Th17 cells. The percentage of Treg cells was increased when EAM mice were treated with iron chelator. The reduced proinflammatory T‐cells resulted in reduced muscle inflammation in the EAM mice. Similar results were found recently that intracellular iron promoted the differentiation of pathogenic T‐cells, including Th1, Th17, and Tfh cells in lupus,^48^ suggesting that the role of iron in pathogenic T‐cells might not restrict to IIM patients but a broader condition with activated inflammatory T‐cells.

PFKFB4 serves as a key regulatory enzyme that maintains cellular levels of fructose 2,6‐bisphosphate to stimulate glycolysis.^49^ In prostate cancer cells, PFKFB4 balanced glycolysis and antioxidant production.^50^ Hif1α activates transcription of *Pfkfb4*, which leads to enhanced glycolysis and increased ATP production in cancer cells.^51^, ^52^ The specific function of PFKFB4 in human T‐cells is not known. Here we found that PFKFB4 was among the most prominent upregulated glycolytic genes in IIM CD4^+^ T‐cells. Iron chelation suppressed AKT‐mTOR signalling and reduced PFKFB4 expression in T‐cells from patients with IIM or HC. CD4^+^ T‐cells from mice treated with iron chelator also showed reduced PFKFB4 expression. In addition, rapamycin inhibited the expression of PFKFB4 in CD4^+^ T‐cells. Knockdown of *Pfkfb4* led to reduced glucose influx into CD4^+^ T‐cells. The changed metabolic programs in CD4^+^ T‐cells by knocking down *Pfkfb4* shifted T‐cell differentiation into Treg cells, whereas the Th1‐ and Th17‐cell differentiations were inhibited.

In summary, we report that iron directed CD4^+^ T‐cells into proinflammatory T‐cells by enhancing glucose metabolism. The data mechanistically link iron to the pathogenicity of CD4^+^ T‐cells in IIM, which have brought new insights into the pathogenesis of IIM. Therapeutic targeting of iron metabolism has the potential to normalize metabolism in CD4^+^ T‐cells and restore proinflammatory CD4^+^ T‐cell phenotype in IIM.

### 3.1 Limitations of the study

Our study mechanistically links iron metabolism to proinflammatory T‐cells in IIM. The limitations of this study which include pathways that promote iron metabolism in CD4^+^ T‐cells in IIM remain undefined. Although our study defines the function of PFKFB4 in helper T‐cell differentiation, the underlying mechanism needs to be addressed in further studies. Broad iron loss has been described to impair energy regulation in muscle cells. Further studies are needed to address the broad effects of iron deficiency on other cell types, including muscle cells.

## 4 MATERIALS AND METHODS

### 4.1 Patients

Patients with IIM who fulfilled 2017 European League Against Rheumatism/American College of Rheumatology Classification Criteria^53^ were recruited from the First Affiliated Hospital, Sun Yat‐sen University. Patients with RA^54^ or SLE^55^ were recruited as disease controls. Patients diagnosed with malignancy, infections or other autoimmune diseases were excluded from this study. Informed consent was acquired from each participant. Age and sex matched HC were recruited from Guangzhou Blood Center. Demographics of the patients and HC are summarized in Tables S1 and S2.

### 4.2 Cell isolation and culture

Peripheral blood mononuclear cells were isolated from freshly collected blood samples by density‐gradient centrifugation. Human total CD4^+^ T‐cells, naïve CD4^+^ T‐cells, human total CD8^+^ T‐cells and mouse CD4^+^ T‐cells were purified using magnetic beads (all from STEMCELL Technologies, Canada). Human T‐cells were cultured and stimulated with anti‐CD3/CD28 beads at a ratio of 1:2.5 for indicated days. Mouse CD4^+^ T‐cells were activated with anti‐mouse CD3 antibody (BioLegend, USA, 2 μg/ml) and anti‐mouse CD28 antibody (BioLegend, USA, 2 μg/ml). Purity of cell population was checked by FACS (>95%). DFX (Sigma‐Aldrich, USA), CPX (MCE, USA), DFO (Sigma‐Aldrich, USA), transferrin (TF, Sigma‐Aldrich, USA) and rapamycin (Selleck Chemicals, USA) were included in some experiments.

### 4.3 Transfection of T‐cells

Electroperforation was performed on human T‐cells as described previously.^41^ Human unstimulated CD4^+^ T‐cells were transfected with *Pfkfb4‐*specific siRNA (120 nM, RiboBio, China) using the P3 Primary Cell 4D‐Nucleofector X Kit L (Lonza, USA). After the transfection, T‐cells were let to rest for 5 h to recover from the electroporation. The knockdown efficiency was validated by western blot.

### 4.4 Flow cytometry

For surface molecules detection, cells were stained with anti‐human PE‐CD3 (Cat 300308), anti‐human FITC‐CD4 (Cat 357406), anti‐human PE/CY7‐CD8 (Cat 344712), anti‐human PE‐CD71 (Cat 334106), anti‐mouse FITC‐CD3 (Cat 100204) and anti‐mouse PE/CY7‐CD8 (Cat 140416) antibodies at 4°C for 30 min. For the determination of Glut1, cells were fixed and permeabilized with the fixation/permeabilization solution (BD Biosciences, USA), followed by anti‐human Alexa Fluor 647‐Glut1 antibody staining (Cat 566580). To measure intracellular cytokine, cells were treated with the cocktail containing of PMA, ionomycin and brefeldin A and the fixation/permeabilization solution, followed by incubation with anti‐human FITC‐IFNγ (Cat 502506) and anti‐human PE‐IL‐17A (Cat 512306) antibodies, or anti‐mouse APC‐IFNγ (Cat 505810), anti‐mouse PE‐IL‐17A (Cat 506904) and anti‐mouse Brilliant Violet 605‐TNFα (Cat 506329) antibodies. To measure the expression of p‐AKT or p‐S6, cells were stained with antibodies against p‐AKT (Ser473, Cat 4060S) or p‐S6 (Ser235/236, Cat 4858S) (both from Cell Signalling Technology, USA), followed by goat anti‐rabbit IgG (Thermo Fisher Scientific, USA). FoxP3 expression was detected in surface‐stained cells after fixation and permeabilization with a FoxP3 Staining Set (eBioscience, USA) using anti‐human PE‐FoxP3 (Cat 320107) and anti‐mouse PE‐FoxP3 (Cat 126404) antibodies.

All the antibodies were from BioLegend (USA) unless indicated. All the FACS samples were analysed by flow cytometer (LSR Fortessa, BD Bioscience, USA). Gating strategies for cells from human and mice were shown in Figure S13.

Cells were labelled with Annexin V‐APC and 7‐AAD (MultiSciences) to assess cell viability by flow cytometry.

### 4.5 T‐cell proliferation

CD4^+^ T‐cells were incubated with carboxyfluorescein succinimidyl ester (5 μM) at 37°C for 15 min. Cells were then diluted with three volumes of pre‐cold culture medium and the reaction was stopped on ice for 5 min. CD4^+^ T‐cell were centrifuged and washed with PBS three times. T‐cells were then activated by anti‐CD3/CD28 beads (Thermo Fisher Scientific, USA) for 4 d. Cell proliferation was assessed by flow cytometry.

### 4.6 Glucose uptake

Glucose uptake was evaluated and analysed by the 2‐NBDG (Thermo Fisher Scientific, USA). Briefly, Total CD4^+^ T‐cells were cultured in glucose‐free RPMI 1640 medium containing 20 μM of 2‐NBDG at 37°C for 30 min. The amount of 2‐NBDG uptake was analysed by flow cytometry.

### 4.7 qPCR

qPCR was performed to measure mRNA expression as we described.^56^ Briefly, total mRNA was extracted using TRIZOL (Invitrogen, USA). Complementary DNA was synthesized with a reverse transcript kit (Accurate Biotechnology, China). The protocol for SYBR Green (Accurate Biotechnology, China) based qPCR was set at an initial denaturation of 30 s, 95°C, followed by 40 cycles of 5 s denaturation at 95°C and 30 s annealing/extension at 60°C. All the target genes were relative to β‐actin. All of the sequences for the primers used in the study are summarized in Table S3.

### 4.8 Western blot

T‐cells were lysed in RIPA Lysis buffer and protein concentrations were determined based on BCA assay (Thermo Fisher Scientific, USA). PAGE‐separated proteins were transferred to a polyvinylidene difluoride membrane (Millipore, Germany) by wet transfer method. Membranes were incubated with the antibodies against HK2 (Santa Cruz, USA, Cat sc‐130358, 1:500), PFKFB4 (Abcam, Hong Kong, Cat ab137785, 1:2000), T‐bet (Abcam, Hong Kong, Cat ab181400, 1:1000), RORg (Thermo Fisher Scientific, USA, Cat 14‐6988‐82, 1:200), p‐AKT (Ser473, Cat 4060S, 1:2000), p‐S6 (S235/236, Cat 4858S, 1:2000), phosphorylation of mTOR (Ser2448, Cat 5536T, 1:2000), mTOR (Cat 2983T, 1:2000), Raptor (Cat 2280T, 1:2000) and β‐actin (Cat 4967S, 1:2000) (all from Cell Signalling Technology, USA) at 4°C overnight. Primary antibodies were identified with horseradish peroxidase‐labelled anti‐rabbit (Cell Signalling Technology, USA, Cat 7074S, 1:2000) or anti‐mouse (Cell Signalling Technology, USA, Cat 7076S, 1:2000) secondary antibodies. Signals were visualized with a Chemilucent Plus Western Blot Enhancing Kit (Millipore, Germany).

### 4.9 Immunohistochemistry

Paraffin sections (5 μm) were dewaxed and treated with 10 min of microwave oven heating with antigen retrieval solution (PH = 6.0, .01 M citrate buffer). Endogenous peroxidase was removed by incubation with 3% hydrogen peroxide. Tissue sections were then incubated with 5% bovine serum albumin, followed by overnight stains with rabbit anti‐human PFKFB4 antibody (1:500) at 4°C. Sections were washed and labelled with anti‐rabbit/mouse GTVision TM + horseradish peroxidase secondary antibody (Gene Tech, China) and visualized with an AEC Detection kit (Abcam, Hong Kong). Then another round of microwave heating was performed to denature any bound antibodies. After that, sections were stained with rabbit anti‐human CD4 primary antibody (Abcam, Hong Kong, Cat ab213215, 1:50) and biotin‐labelled Goat Anti‐ rabbit /mouse IgG (Boster Biological Technology, China), followed by streptavidin–biotin complex containing alkaline phosphatase (Boster Biological Technology, China). Finally, sections were visualized with an NBT/BCIP Detection Kit (Abcam, Hong Kong).

### 4.10 Seahorse assay

Immunometabolism was measured using the Cell Mito Stress Test Kit or the Glycolysis stress kit, respectively (Agilent, USA). CD4^+^ T‐cells were plated at .2 million cells per well in a Seahorse XF96 plate (Agilent, USA). Cells were let to attach to the plate using Cell‐Tak (Corning, USA). To measure mitochondrial respiration, the T‐cells were sequentially treated with oligomycin (1.5 mM), carbonyl cyanide‐*p*‐trifluoromethoxyphenylhydrazone (1.5 mM) and rotenone/antimycin A (1.0 mM). To measure glycolytic activity, the cells were subsequentially treated with glucose (10 mM), oligomycin (1 μM) and 2‐DG (50 mM).

### 4.11 RNA‐seq

CD4^+^ T‐cells from HC were stimulated with anti‐CD3/CD28 beads. DFX or transferrin was included to some of the experiments for 3 d. RNA libraries were sequenced on a BGIseq‐500 platform (BGI‐Shenzhen, China) to generate single‐end 50 bases reads. Reads were aligned against human reference genome (GRCh38) using HISAT2 (version 2.2.1). By using featureCounts (version 2.0.1), read counts of gene were further quantified. Genes with more than 10 reads in total across all samples were adopted for further analysis. Sva package was used for the removal of batch effects by applying ComBat‐seq function.^57^ Differential gene expression in different conditions was analysed using DESeq2 (version 1.28.1) R package^58^ via a moderated *t*‐test of Benjamini–Hochberg method. The cut‐off values for significantly DEGs were set as adjusted‐*p* < .01 and |log2(foldchange)| > .05. clusterProfiler^59^ (version 4.1.0) R package was used to assign biological functions to the DEGs, including GO, KEGG, RPD and WP signalling pathways. GSEA^60^ was also applied to evaluate signalling pathways enriched in different treatment group, respectively. Normalized enrichment score was used for the estimation of enrichment. Enriched signalling pathway was set at the threshold of *p* < .05. Data regarding RNA‐seq analysis were analysed and visualized using R (version 4.0.5) and RStudio (integrated development for R; RStudio).

### 4.12 Experimental autoimmune myositis (EAM) model

Female BALB/c mice were purchased from Guangdong Medical Laboratory Animal Center. For EAM induction, 6‐week‐old mice were immunized with 100 μl of 50% complete Freund's adjuvant (Sigma, USA) containing 1‐mg myosin (or with an equal volume of PBS in control group) on bilateral sides of the hind foot pads, the tail base and flanks four times at 1‐week intervals as previously described.^61^ Before the first immunization, 500‐ng pertussis toxin (MCE, USA) and an equal volume of saline were injected intraperitoneally in modelling group and control group, respectively.

An iron chelator DFX was used in this study and diluted in vehicle (1% DMSO in saline) solution. EAM mice were randomized into DFX group and vehicle group (*n* = 6). Mice were fed with DFX (30 mg/kg/d) or an equal volume of vehicle in the water bottle after the second immunization for 2 weeks. Additionally, EAM mice were treated with rapamycin (1.5 mg/kg) or equal volume of vehicle (*n* = 6) intraperitoneally daily after the second immunization for 2 weeks. Muscle strengths were assessed using an inverted screen test as previous stated.^62^ After treatment, quadriceps muscles were collected for hematoxylin and eosin staining and qPCR. Sizes of spleens were evaluated. Lymph nodes were obtained.

### 4.13 Statistics

Statistical analyses were performed using GraphPad Prism 8.0. All data are expressed as mean ± SEM and *p* < .05 was considered significant.

## CONFLICT OF INTEREST

The authors have declared that no conflict of interest exists.

## Supporting information

Supporting Information

## Data Availability

The RNA‐Seq data that support the findings of this study have been deposited in the Genome Sequence Archive (Genomics, Proteomics & Bioinformatics 2021) in National Genomics Data Center (Nucleic Acids Res 2022), China National Center for Bioinformation / Beijing Institute of Genomics, Chinese Academy of Sciences (GSA‐Human: HRA002731) that are publicly accessible at https://ngdc.cncb.ac.cn/gsa-human.
