# Supplementary material for: Iron controls T helper cell pathogenicity by promoting glucose metabolism in autoimmune myopathy
Source: Clin Transl Med. 2022 Aug 2;12(8):e999. doi: 10.1002/ctm2.999 (PMC9345506; doi:10.1002/ctm2.999)
Supplement: Supplementary file 1 — Supporting Information [file CTM2-12-e999-s001.zip › Suplementary file revision_clean copy.docx]

| S. Table 1. **Demographics of DM/PM patients in this study**. | | | |  |
| --- | --- | --- | --- | --- |
| **Parameters** | **DM (n=44)** | **PM (n=19)** | **References** | |
| Age, year, median (min–max) | 48 (14-83) | 47 (20-70) |  | |
| Female/male (no.) | 29/15 | 15/4 |  | |
| Disease duration, year | 1.6 ± 0.3 | 2.1 ± 0.6 |  | |
| **Clinical parameters** |  |  |  | |
| CRP, mg/L | 9.3 ± 1.4 | 24.2 ± 12.3 | 0.00-3.00 | |
| ESR, mm/h | 33.3 ± 3.2 | 31.8 ± 5.5 | ≤ 20 | |
| CK, U/L | 218.6 ± 61.3 | 1770.3 ± 552.0 | 25-200 | |
| AST, U/L | 50.7 ± 8.2 | 97.5 ± 23.9 | 1-37 | |
| ALT, U/L | 44.1 ± 4.9 | 77.4 ± 14.6 | 1-40 | |
| LDH, U/L | 297.0 ± 17.3 | 580.4 ± 115.8 | 114-240 | |
| Ferritin, μg/L | 617.9 ± 111.1 | 414.1 ± 79.6 | 16.40-323.00 | |
| **IIM autoantibody spectrum**^†^ |  |  |  | |
| anti-MDA5, P/N, no. | 18/21 | 0/16 | negative | |
| anti-PL7, P/N, no. | 2/37 | 1/15 | negative | |
| anti-PL12, P/N, no. | 2/37 | 2/14 | negative | |
| anti-EJ, P/N, no. | 2/37 | 2/14 | negative | |
| anti-OJ, P/N, no. | 0/39 | 0/16 | negative | |
| anti-Jo-1, P/N, no. | 7/32 | 6/10 | negative | |
| anti-SRP, P/N, no. | 0/39 | 4/12 | negative | |
| anti-HMGCR, P/N, no. | 1/38 | 1/15 | negative | |
| anti-NXP2, P/N, no. | 0/39 | 1/15 | negative | |
| anti-Mi-2β, P/N, no. | 1/38 | 0/16 | negative | |
| anti-TIF1γ, P/N, no. | 5/34 | 1/15 | negative | |
| anit-PM-Scl75, P/N, no. | 1/38 | 2/14 | negative | |
| anti-Ro-52, P/N, no. | 15/24 | 2/14 | negative | |
| **Medication** |  |  |  | |
| Glucocorticoids, n (%) | 42 (95) | 18 (95) |  | |
| Mycophenolate mofetil, n (%) | 12 (27) | 8 (42) |  | |
| Cyclophosphamide, n (%) | 12 (27) | 4 (21) |  | |

Note: All data are expressed as mean ± SEM. DM, dermatomyositis. PM, Polymyositis.

^†^Autoantibody spectrum was available in 39 of the recruited DM patients and 16 of the recruited PM patients.

S. Table 2. **Demographics of healthy and disease controls in this study**.

| **Parameters** | **HC (n=63)** | **SLE (n=13)** | **RA (n=15)** | **References** |
| --- | --- | --- | --- | --- |
| Age, year, median (min–max) | 48 (14-83) | 34 (13-68) | 62 (33-71) |  |
| Female/male (no.)  Disease duration, year | 44/19  − | 10/3  7.2 ± 1.9 | 11/4  8.2 ± 2.0 |  |
| CRP, mg/L | − | 5.9 ± 1.8 | 30.8 ± 10.9 | 0.00-3.00 |
| ESR, mm/h | − | 30.5 ± 6.2 | 49.6 ± 8.9 | ≤ 20 |
| ANA, U/ml | − | 175.8 ± 67.0 | − | 0.00-12.00 |
| ani-ds-DNA antibody, IU/ml | − | 43.8 ± 16.6 | − | 0.00-30.00 |
| C3, g/L | − | 0.6 ± 0.0 | − | 0.79-1.17 |
| C4, g/L | − | 0.2 ± 0.0 | − | 0.17-0.31 |
| proteinuria, P/N, no. | − | 5/8 | − | negative |
| ALB, g/L | − | 32.6 ± 2.2 | − | 35-50 |
| creatinine, μmol/L | − | 101.9 ± 21.6 | − | 53-115 |
| RF, Ku/L | − |  | 240.4 ± 44.1 | 0.00-20.00 |
| Anti-CCP antibody, U/ml | − |  | 28.9 ± 6.6 | 0.00-5.00 |

Note: All data are expressed as mean ± SEM. HC, healthy controls, SLE, systemic lupus erythematosus. RA, rheumatoid arthritis.

S. Table 3. **Human and mouse PCR primers used in this study**.

| **Gene** | **Forward primer** | **Reverse primer** |  |  |  |
| --- | --- | --- | --- | --- | --- |
| Human *Glut1* | GGCCAAGAGTGTGCTAAAGAA | ACAGCGTTGATGCCAGACAG |  |  |  |
| Human *Pfk1* | GGTGCCCGTGTCTTCTTTGT | AAGCATCATCGAAACGCTCTC |  |  |  |
| Human *Ldha* | ATGGCAACTCTAAAGGATCAGC | CCAACCCCAACAACTGTAATCT |  |  |  |
| Human *Hk2* | GAGCCACCACTCACCCTACT | CCAGGCATTCGGCAATGTG |  |  |  |
| Human *Pkm2* | ATGTCGAAGCCCCATAGTGAA | TGGGTGGTGAATCAATGTCCA |  |  |  |
| Human *Pfkfb4* | CAACATCGTGCAAGTGAAACTG | GACTCGTAGGAGTTCTCATAGCA |  |  |  |
| Human *Gapdh* | GGAGCGAGATCCCTCCAAAAT | GGCTGTTGTCATACTTCTCATGG |  |  |  |
| Human *c-Myc* | GGCTCCTGGCAAAAGGTCA | CTGCGTAGTTGTGCTGATGT |  |  |  |
| Human *Hif1α* | ATCCATGTGACCATGAGGAAATG | TCGGCTAGTTAGGGTACACTTC |  |  |  |
| Human *REDD1* | TGAGGATGAACACTTGTGTGC | CCAACTGGCTAGGCATCAGC |  |  |  |
| Human *Bnip3* | CAGGGCTCCTGGGTAGAACT | CTACTCCGTCCAGACTCATGC |  |  |  |
| Human *β-actin* | CATGTACGTTGCTATCCAGGC | CTCCTTAATGTCACGCACGAT |  |  |  |
| Mouse *Il10* | CTTACTGACTGGCATGAGGATCA | GCAGCTCTAGGAGCATGTGG |  |  |  |
| Mouse *Il17a* | TCAGCGTGTCCAAACACTGAG | CGCCAAGGGAGTTAAAGACTT |  |  |  |
| Mouse *Il21* | GGGGACAGTGGCCCATAAATC | GTGCCCCTTTACATCTTGTGG |  |  |  |
| Mouse *Tnfα* | CAGGCGGTGCCTATGTCTC | CGATCACCCCGAAGTTCAGTAG |  |  |  |
| Mouse *Ifng* | TTACTGCCACGGCACAGTC | AGATAATCTGGCTCTGCAGG |  |  |  |
| Mouse *β-actin* | GGCTGTATTCCCCTCCATCG | CCAGTTGGTAACAATGCCATGT |  |  |  |


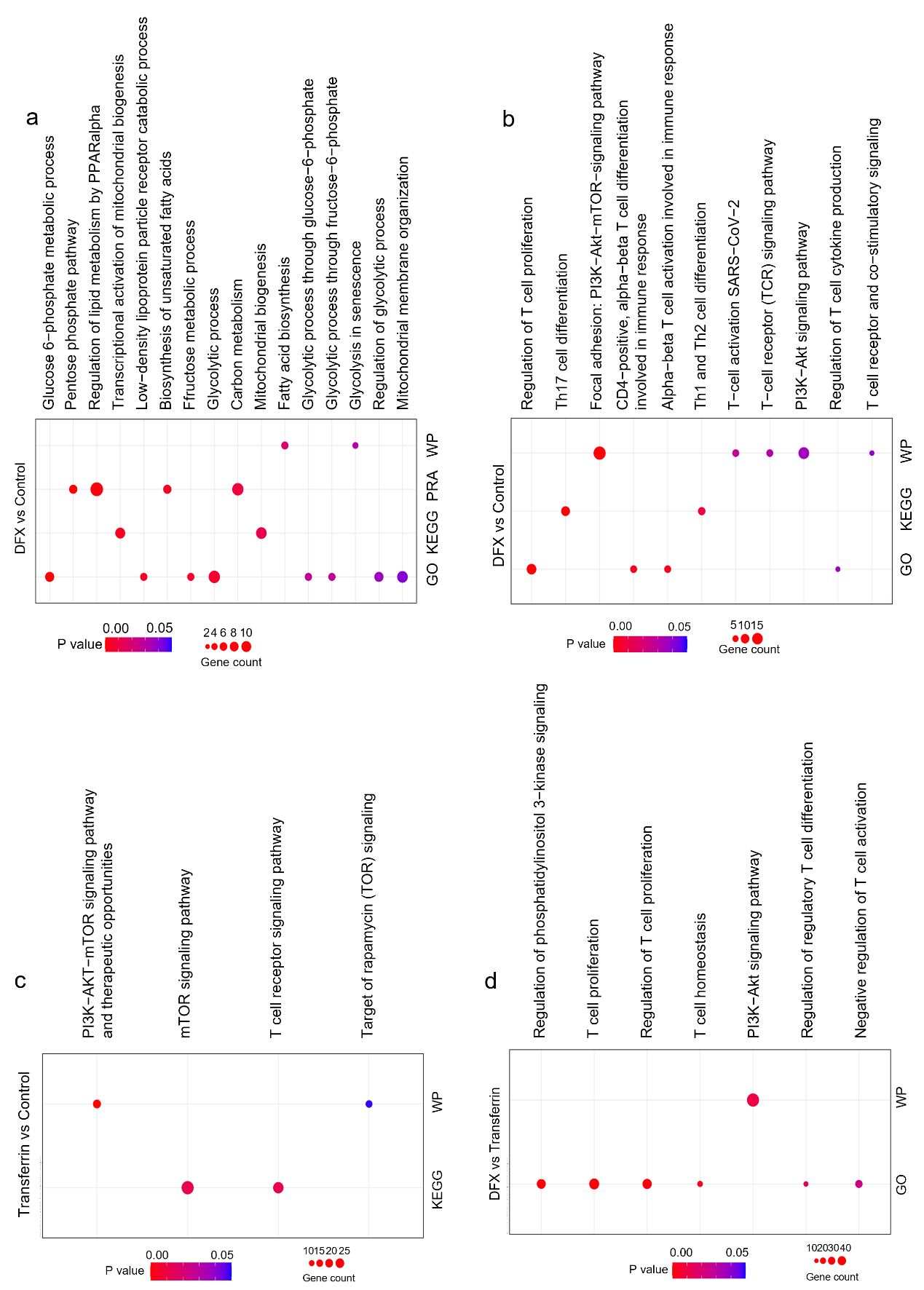


S. Figure 1. **Enriched signaling pathways derived from DEGs demonstrated metabolism and T cell activation pathways in different conditions**. DEGs were identified from differential gene expression analysis using DESeq2 method. Functional signaling pathways including metabolism and T cell activation pathways, were further enriched by using Clusterprofiler method. The dotplot illustrated p-value and DEGs of pathways under Deferasirox (DFX) versus Control (a), DFX versus Control (b), Transferrin (TF) versus Control (c) and DFX versus TF conditions (d). The dot size was corresponding to the number of DEGs and the color corresponding to the p-value. (GO: Gene ontology; KEGG: Kyoto Encyclopedia of Genes and Genomes; RPD: Reactome Pathway Database; WP: WikiPathways).


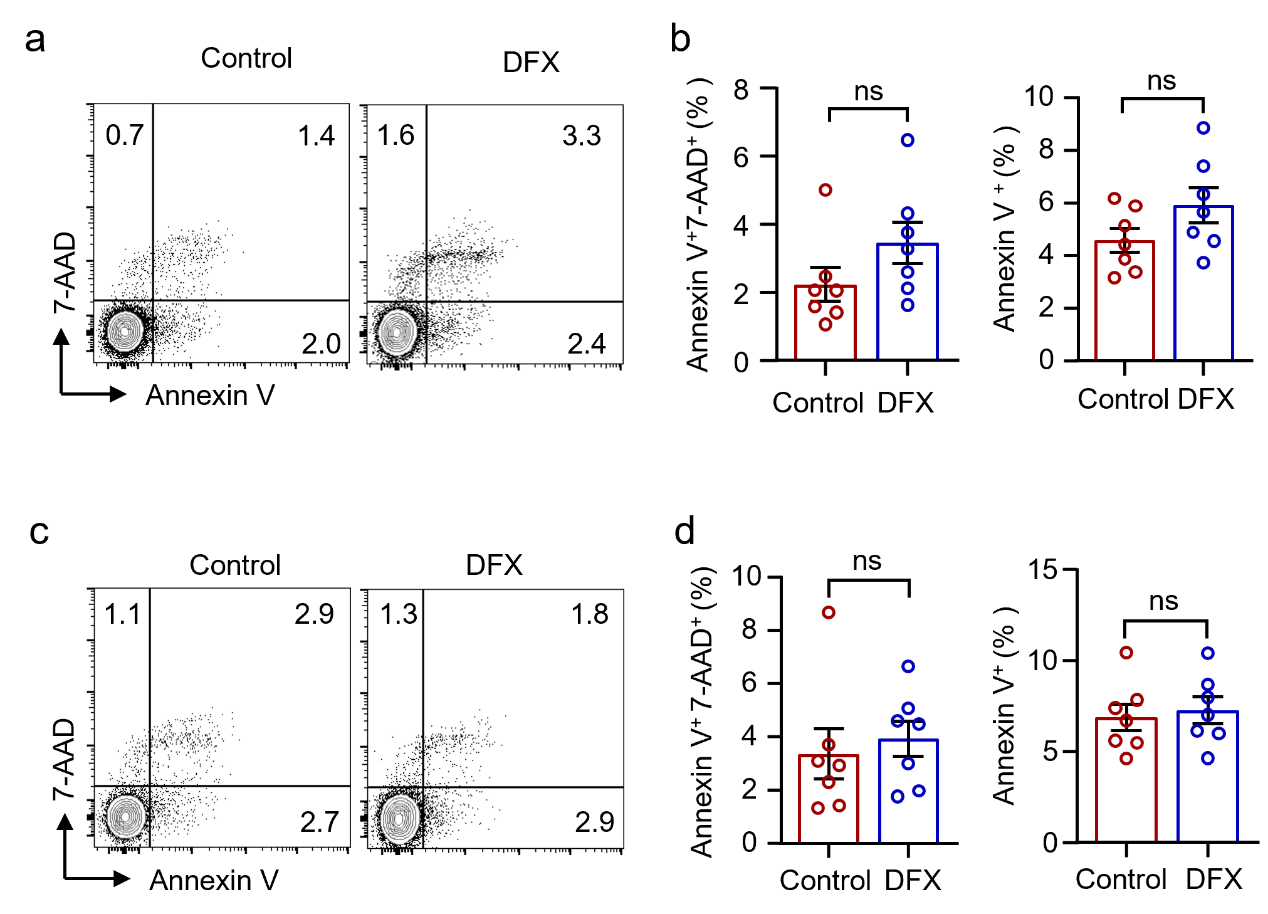


S. Figure 2. **Iron chelation did not induce T cell death**. CD4^+^ T cells isolated from Healthy PBMCs were stimulated with anti-CD3/CD28 beads in the presence or absence of DFX (2μM) for 3 days. Cell death were determined by Annexin V and 7-AAD. (a) Representative counter plots of CD4^+^ T cells. (b) quantification of the frequency of Annexin V^+^7-AAD^+^ and Annexin V^+^ cells in CD4^+^ T cells. (c) Representative counter plots of CD8^+^ T cells. (d) quantification of the frequency of Annexin V^+^7-AAD^+^ and Annexin V^+^ cells in CD8^+^ T cells. Data from 7 independent samples. Data are mean ± SEM. ns: not significant.


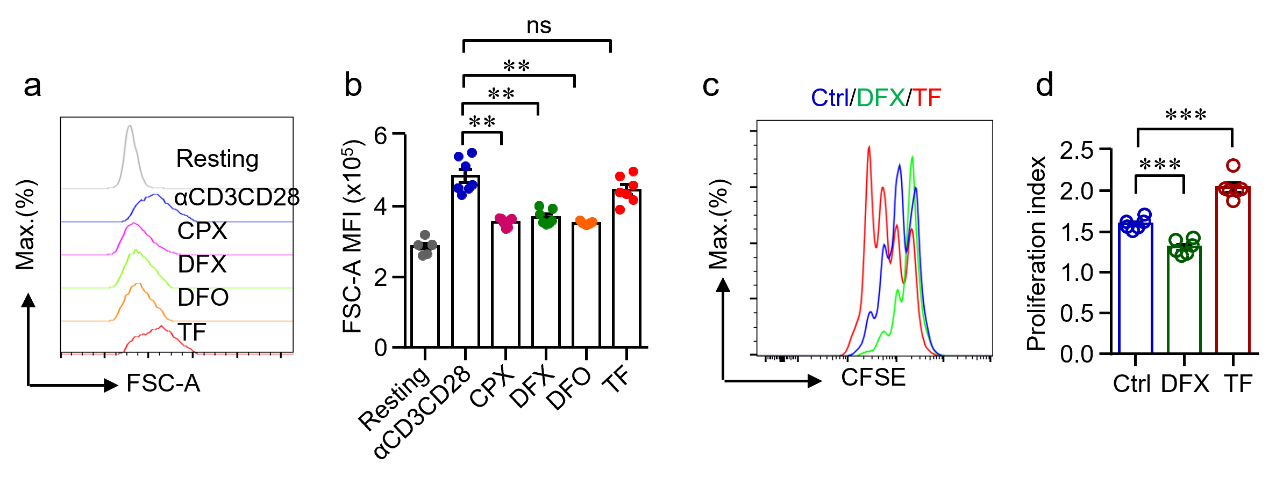


S. Figure 3. **Iron chelation inhibits CD4^+^ T cell proliferation**. CD4^+^ T cells isolated from healthy PBMCs were stimulated with anti-CD3/CD28 beads. Ciclopirox (CPX, 1μM), DFX (2μM), Deferoxamine (DFO, 2μM) and TF (50μg/ml) was included in some of the experiments. (a, b) Cell size of CD4^+^ T cells was measured by flow cytometry (n=7). (c, d) CD4^+^ T cells were labeled with CFSE and cultured for 3 days. Cell proliferation was measured by flow cytometry. Representative histograms were shown. Proliferation index was calculated in DFX, TF or Ctrl treated CD4^+^ T cells (n=6). All data are mean ± SEM. **p<0.01 and ***p<0.001 by one-way ANOVA followed by adjustments for multiple comparisons. ns: not significant.


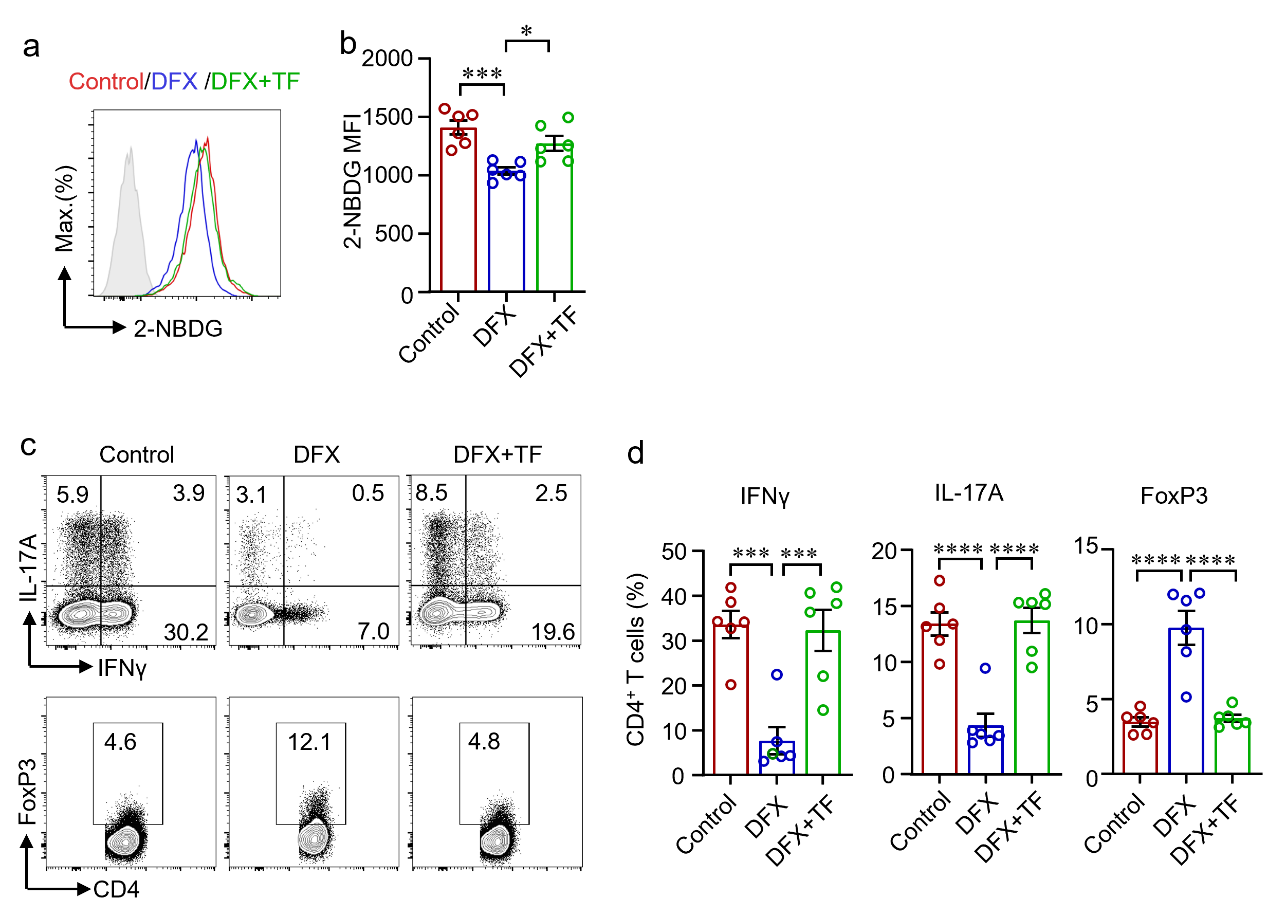


S. Figure 4. **Transferrin reversed the inhibitory effects of iron chelation on CD4^+^ T cells.** (a, b) CD4^+^ T cells isolated from healthy PBMCs were stimulated with anti-CD3/CD28 beads in the presence or absence of DFX (2μM) and TF (50μg/ml) for 3 days. CD4^+^ T cells were incubated with 2-NBDG (glucose analog) and glucose uptake was accessed by flow cytometry for the uptake of 2-NBDG. Mean fluorescence intensity (MFI) was shown. (c, d) CD4^+^ T cells isolated from healthy PBMCs were stimulated with anti-CD3/CD28 beads in the presence or absence of DFX (2μM) and TF (50μg/ml) for 5 days. Representative counter plots for IFNγ, IL-17A and FoxP3 expression in CD4^+^ T cells as measured by flow cytometry. Data from 6 independent samples. Data are mean ± SEM. *p<0.05, ***p<0.001, ****p<0.0001 by one-way ANOVA followed by adjustments for multiple comparisons.


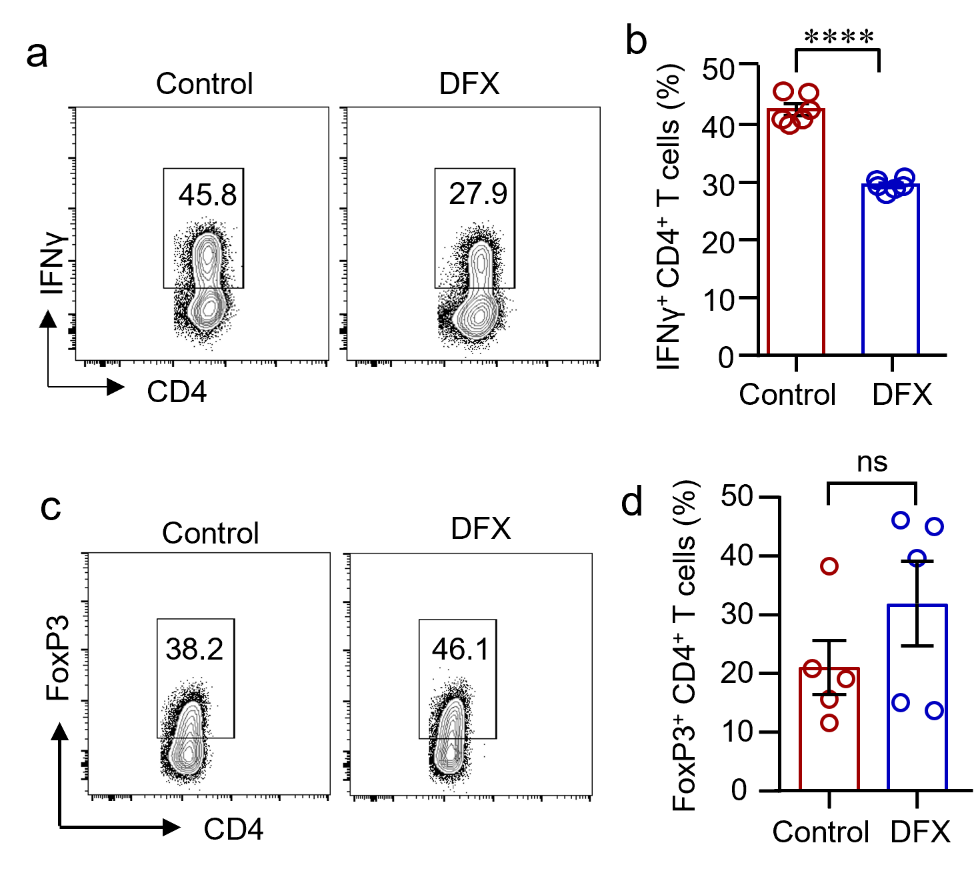


S. Figure 5. **Iron chelation inhibited Th1 differentiation.** (a, b) Naïve CD4^+^ T cells were isolated from healthy PBMCs and cultured in Th1 cell differentiation condition (anti-CD3/CD28 beads, 10ng/ml of recombinant IL-12 and 1μg/ml of anti-human IL-4 antibody) in the presence or absence of DFX (2μM) for 5 days. IFNγ production was measured by flow cytometry (n=6). Representative counter plots were shown. (c, d) Naïve CD4^+^ T cells were isolated from healthy PBMCs and cultured in Treg cell differentiation condition (anti-CD3/CD28 beads, 10ng/ml of TGF-β1) in the presence or absence of DFX (2μM) for 5 days. FoxP3 expression was measured by flow cytometry (n=5). Representative counter plots were shown. Data are mean ± SEM. ****p<0.0001 by one-way ANOVA followed by adjustments for multiple comparisons. ns: not significant.


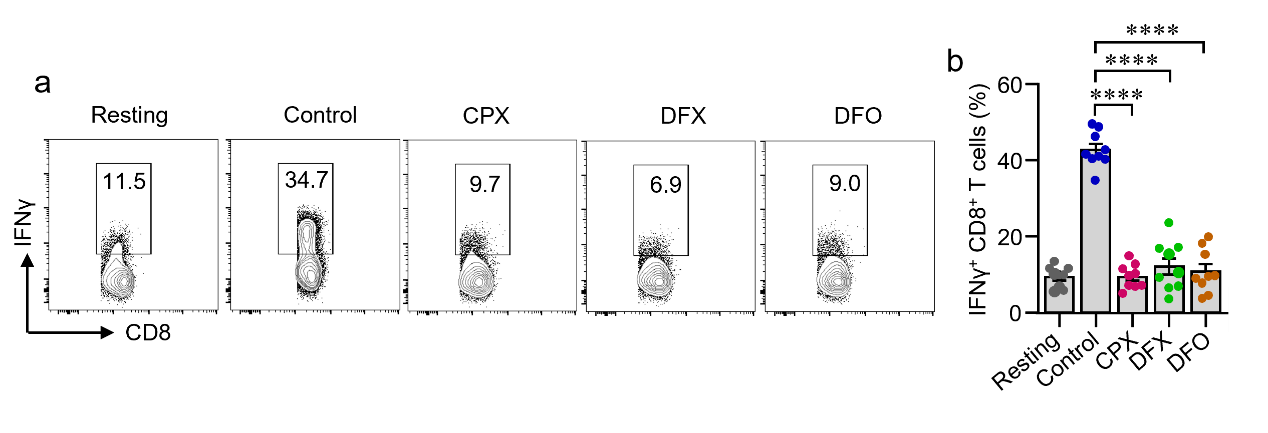


S. Figure 6. **Iron chelation suppressed IFNγ production in CD8^+^ T cells.** CD8^+^ T cells isolated from Healthy PBMCs were stimulated with anti-CD3/CD28 beads in the presence or absence of CPX (1μM), DFX (2μM) and DFO (2μM) for 5 days. (a) Representative counter plots for IFNγ expression in CD8^+^ T cells as measured by flow cytometry. (b) quantification of the frequency of CD8^+^IFNγ^+^ cells for 8 independent samples. Data are mean ± SEM. ****p<0.0001 by one-way ANOVA followed by adjustments for multiple comparisons.


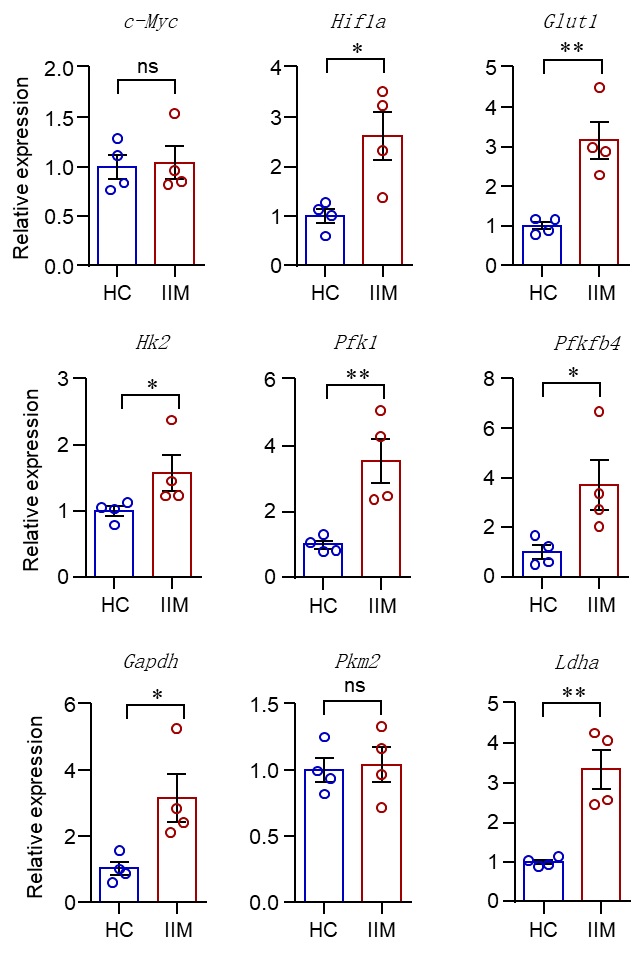


S. Figure 7. **Enhanced** g**lycolytic gene expression in CD4^+^ T cells from patients with IIM**. CD4^+^ T cells were isolated from patients with idiopathic inflammatory myopathies (IIM) or healthy controls (HC). Gene expression in CD4^+^ T cells was measured by qPCR (n=4). All data are mean ± SEM. *p<0.05, **<0.01 by Student’s t test. ns: not significant.


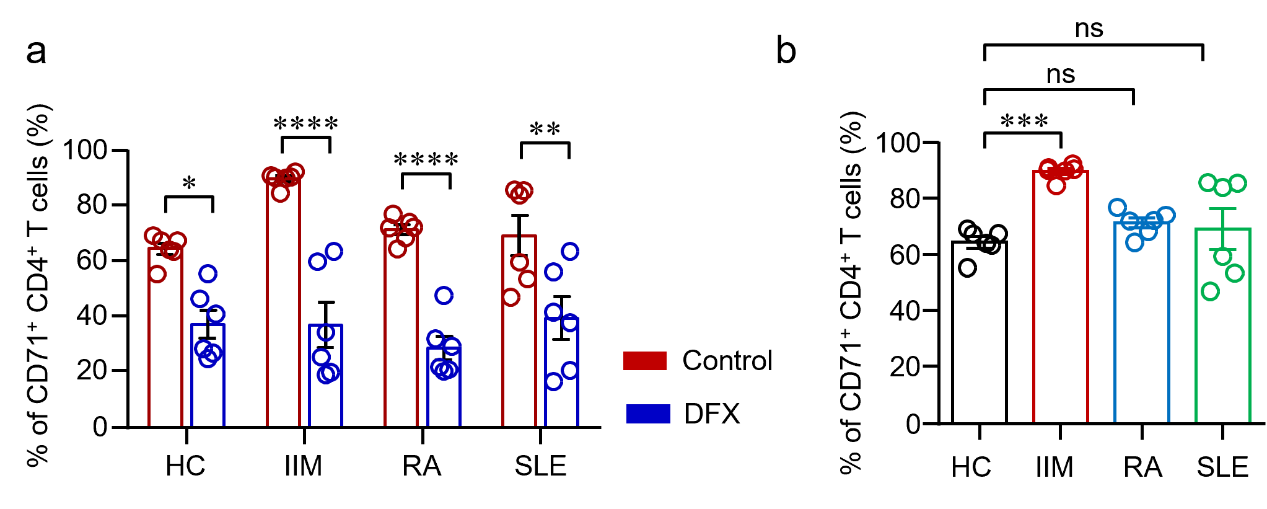


S. Figure 8. **Iron chelation decreased CD71 expression in CD4^+^ T cells**. CD4^+^ T cells from patients with IIM, rheumatoid arthritis (RA) and systemic lupus erythematosus (SLE) or HC were stimulated with anti-CD3/CD28 beads for 3 days. (a) CD71 expression in CD4^+^ T cells cultured in presence or absence of DFX was analyzed by flow cytometry. (b) CD71 expression in CD4^+^ T cells from patients with IIM, RA and SLE and HC was analyzed by flow cytometry. n=6 in each group. Data are mean ± SEM. *p<0.05, **p<0.01, ***p<0.001, ****p<0.0001 by Student’s t test in a and one-wan ANOVA followed by multiple test adjustment in b. ns: not significant.


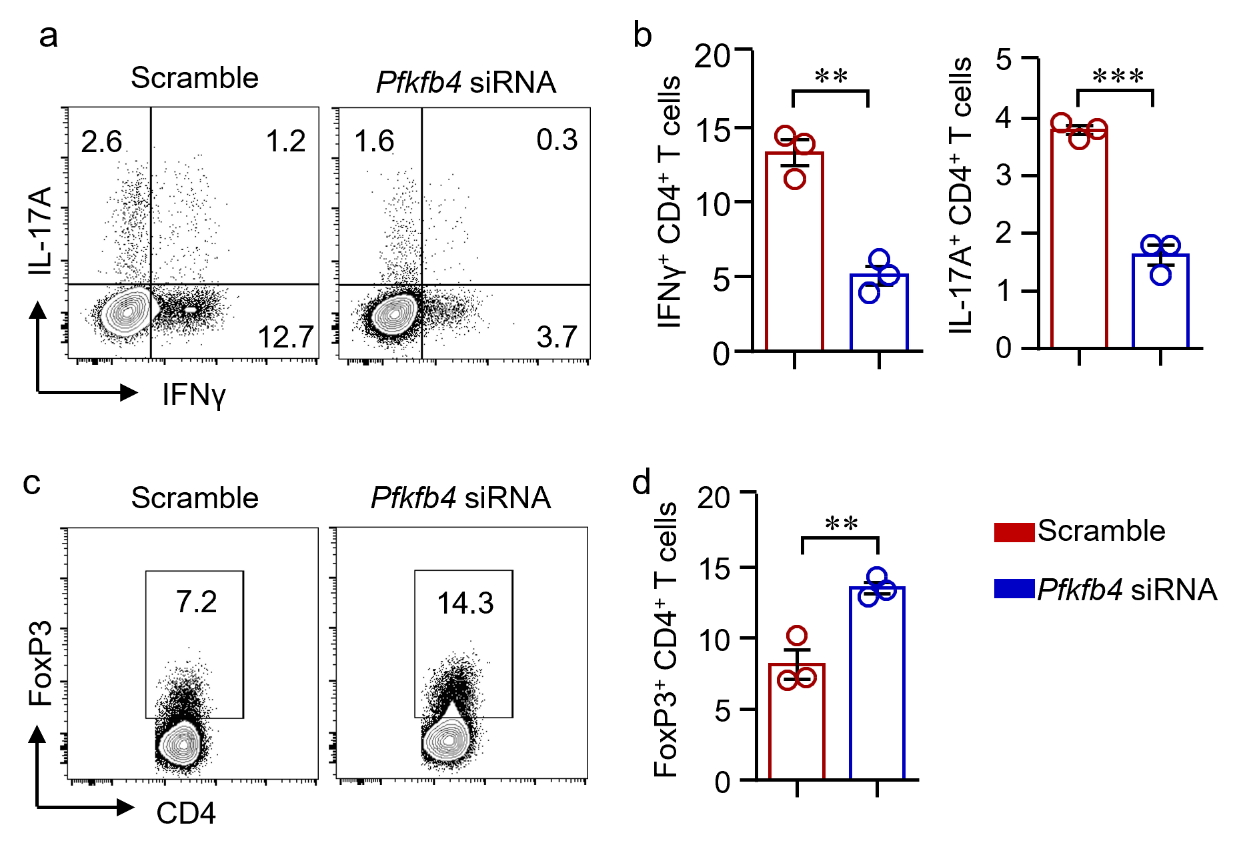


S. Figure 9. **PFKFB4 regulated CD4^+^ T cell differentiation**. *Pfkfb4* was knocked down in CD4^+^ T cells from patients with IIM. CD4^+^ T cells were stimulated with anti-CD3/CD28 beads for 5 days. (a, c) Representative counter plots for IFNγ, IL-17A and FoxP3 expression in CD4^+^ T cells. (b) quantification of IFNγ^+^CD4^+^ T cells, IL-17A^+^CD4^+^ T cells, and (d) FoxP3^+^CD4^+^ T cells were measured by flow cytometry. Data from 3 independent samples. Data are mean ± SEM. **p<0.01, ***p<0.001 by Student’s t test.


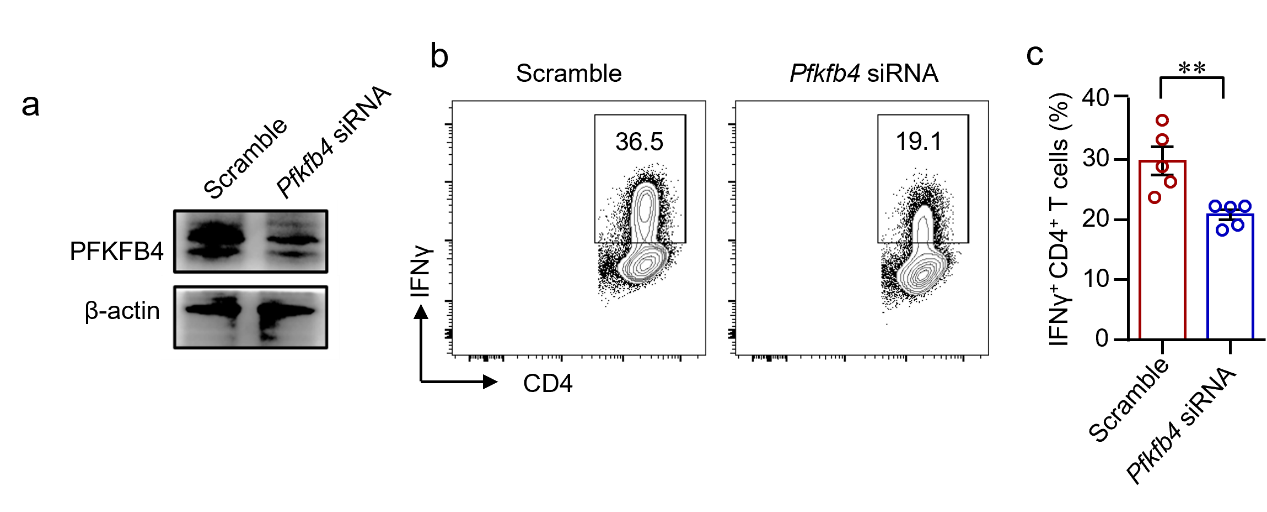


S. Figure 10. **PFKFB4 is required during Th1 differentiation**. Naïve CD4^+^ T cells were isolated from healthy PBMCs and treated with *Pfkfb4* siRNA or scramble siRNA by electroperforation. Cells were cultured in Th1 cell differentiation condition (anti-CD3/CD28 beads, 10ng/ml of recombinant IL-12 and 1μg/ml of anti-human IL-4 antibody) for 5 days. (a) Pfkfb4 knockdown efficiency was confirmed by western blot. (b-c) IFNγ production was measured by flow cytometry. Representative counter plots were shown and data from 5 independent samples. Data are mean ± SEM. **p<0.01 by Student’s t test.


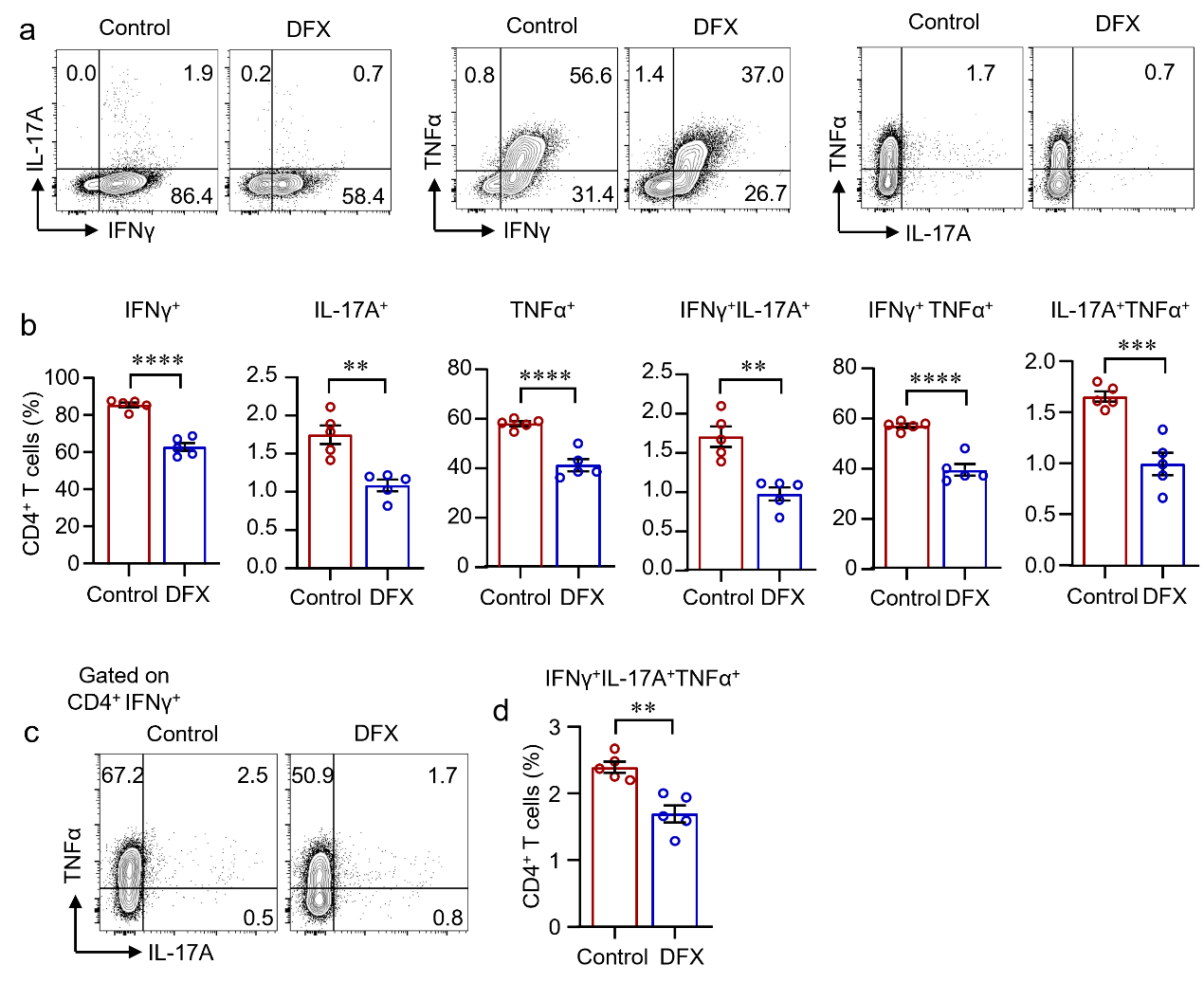


S. Figure 11. **Iron chelation suppressed multifunctional CD4^+^ T cells in experimental autoimmune myositis**. Splenic cells from experimental autoimmune myositis (EAM) mice were cultured with anti-CD3 antibody and anti-CD28 antibody in presence or absence of DFX (2μM) for 3 days. (a) Representative counter plots for IFNγ, IL-17A and TNFα expression in CD4^+^ T cells. (b) quantification of IFNγ^+^, IL-17A^+^, TNFα^+^, IFNγ^+^IL-17A^+^, IFNγ^+^ TNFα^+^and IL-17A^+^TNFα^+^ cells in CD4^+^ T cells. (c, d) quantification of IFNγ^+^ IL-17A^+^TNFα^+^CD4^+^ T cells measured by flow cytometry. Representative counter plots were shown. Data from 5 independent samples. Data are mean ± SEM. **p<0.01, ***p<0.001, ****p<0.0001 by Student’s t test.


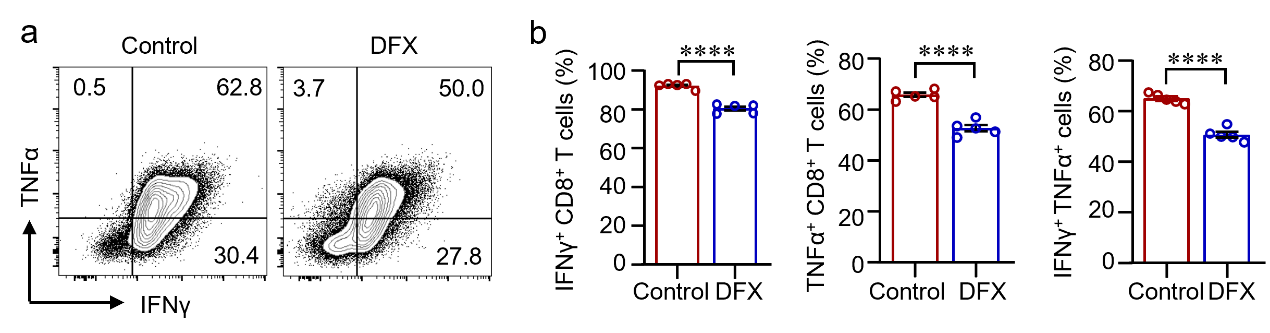


S. Figure 12. **Iron chelation reduced cytokine production in CD8^+^ T cells in experimental autoimmune myositis.** (a, b) IFNγ and TNFα production in CD8^+^ T cells from experimental autoimmune myositis (EAM) treated with DFX or vehicle was measured by flow cytometry. Representative counter plots were shown and data from 5 independent samples. Data are mean ± SEM. ****p<0.0001 by Student’s t test.


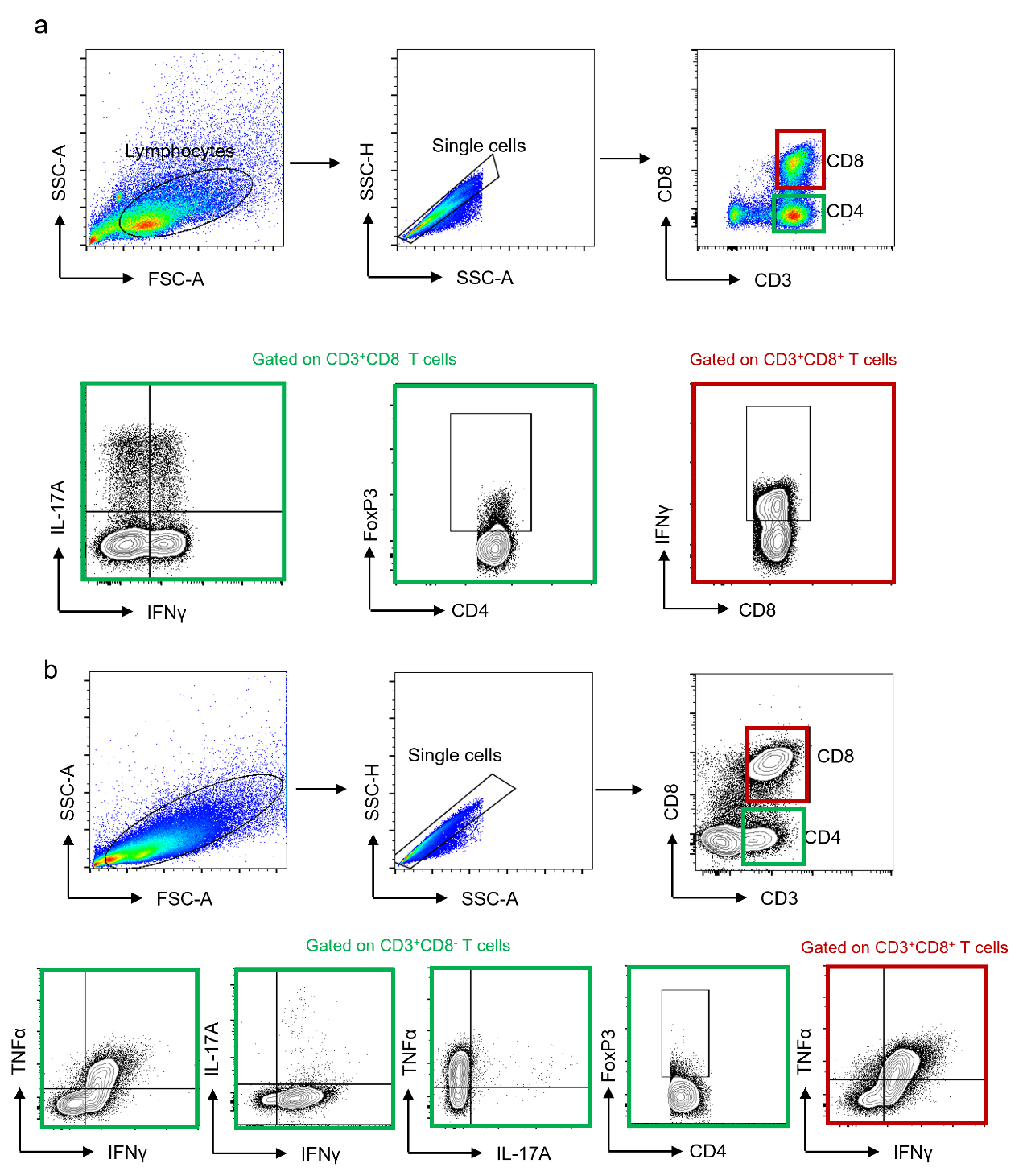


S. Figure 13. (a) Gating strategy for human PBMC analysis. (b) Gating strategy of mouse cell analysis.
