# Supplementary figures and images for: Iron controls T helper cell pathogenicity by promoting glucose metabolism in autoimmune myopathy
Source: Clin Transl Med. 2022 Aug 2;12(8):e999. doi: 10.1002/ctm2.999 (PMC9345506; doi:10.1002/ctm2.999)

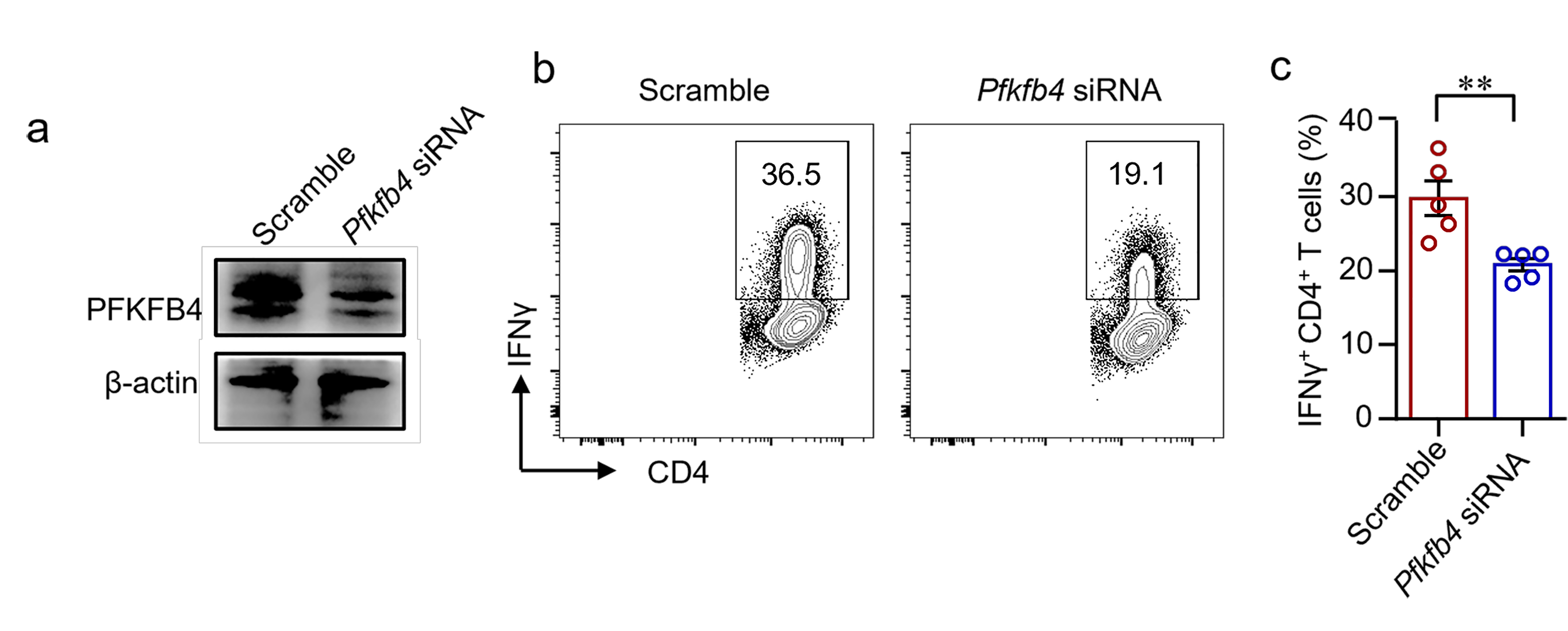

Supplement: Supplementary file 1 — Supporting Information [file CTM2-12-e999-s001.zip › Figure_10_supplnfo.docx]

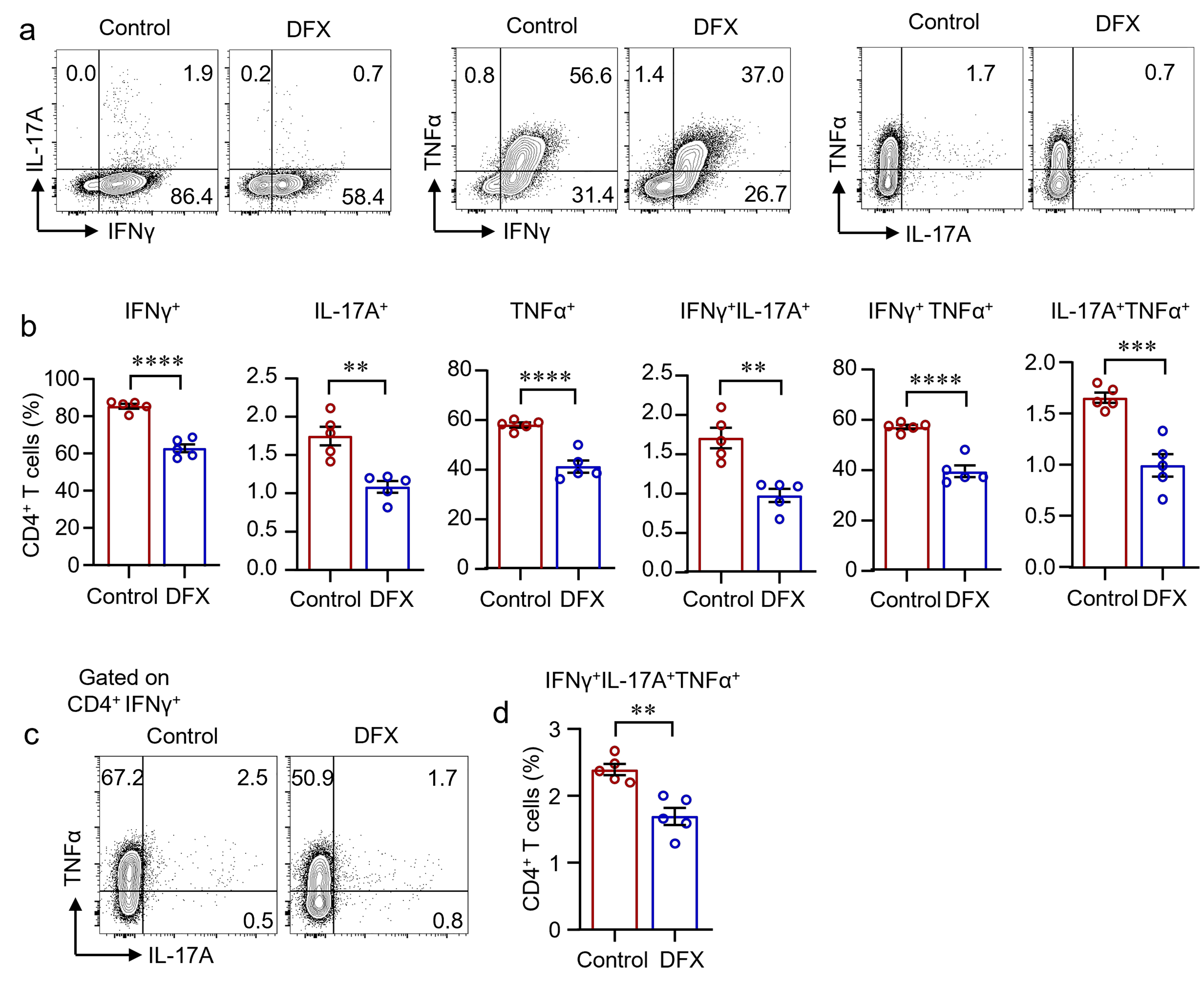

Supplement: Supplementary file 1 — Supporting Information [file CTM2-12-e999-s001.zip › Figure_11_supplnfo.docx]

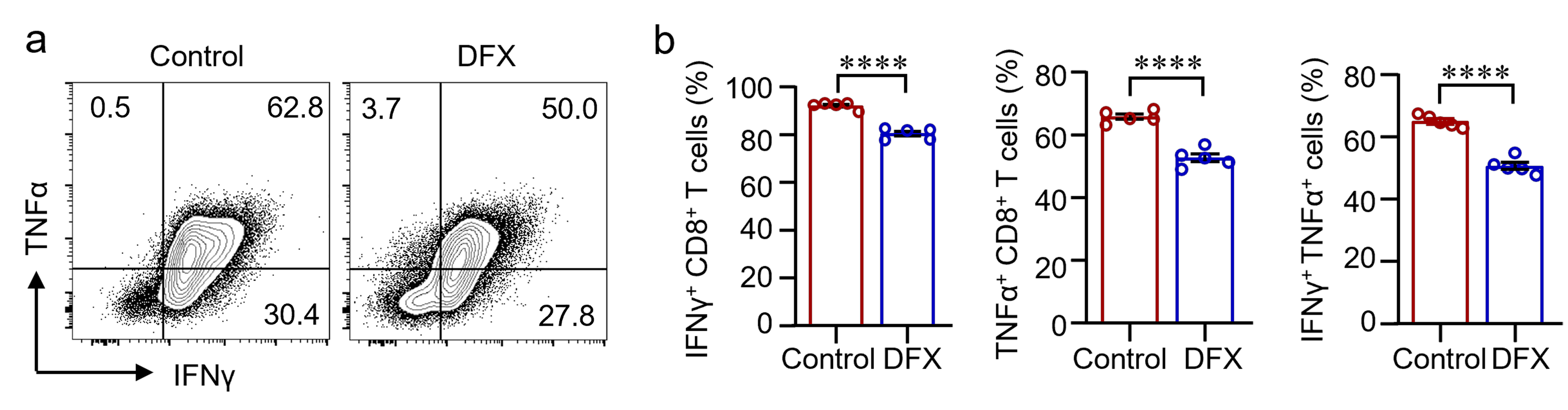

Supplement: Supplementary file 1 — Supporting Information [file CTM2-12-e999-s001.zip › Figure_12_supplnfo.docx]

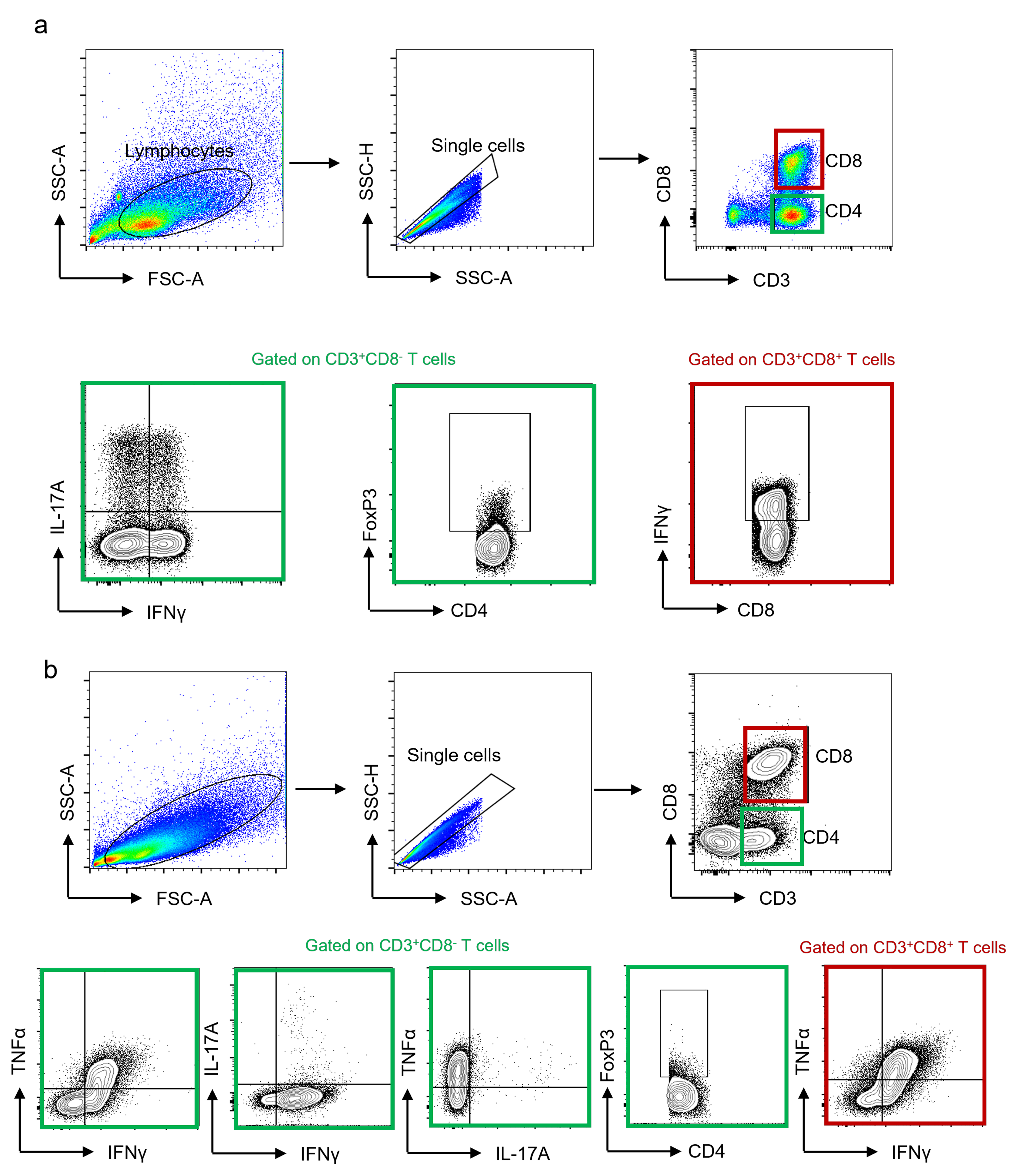

Supplement: Supplementary file 1 — Supporting Information [file CTM2-12-e999-s001.zip › Figure_13_supplnfo.docx]

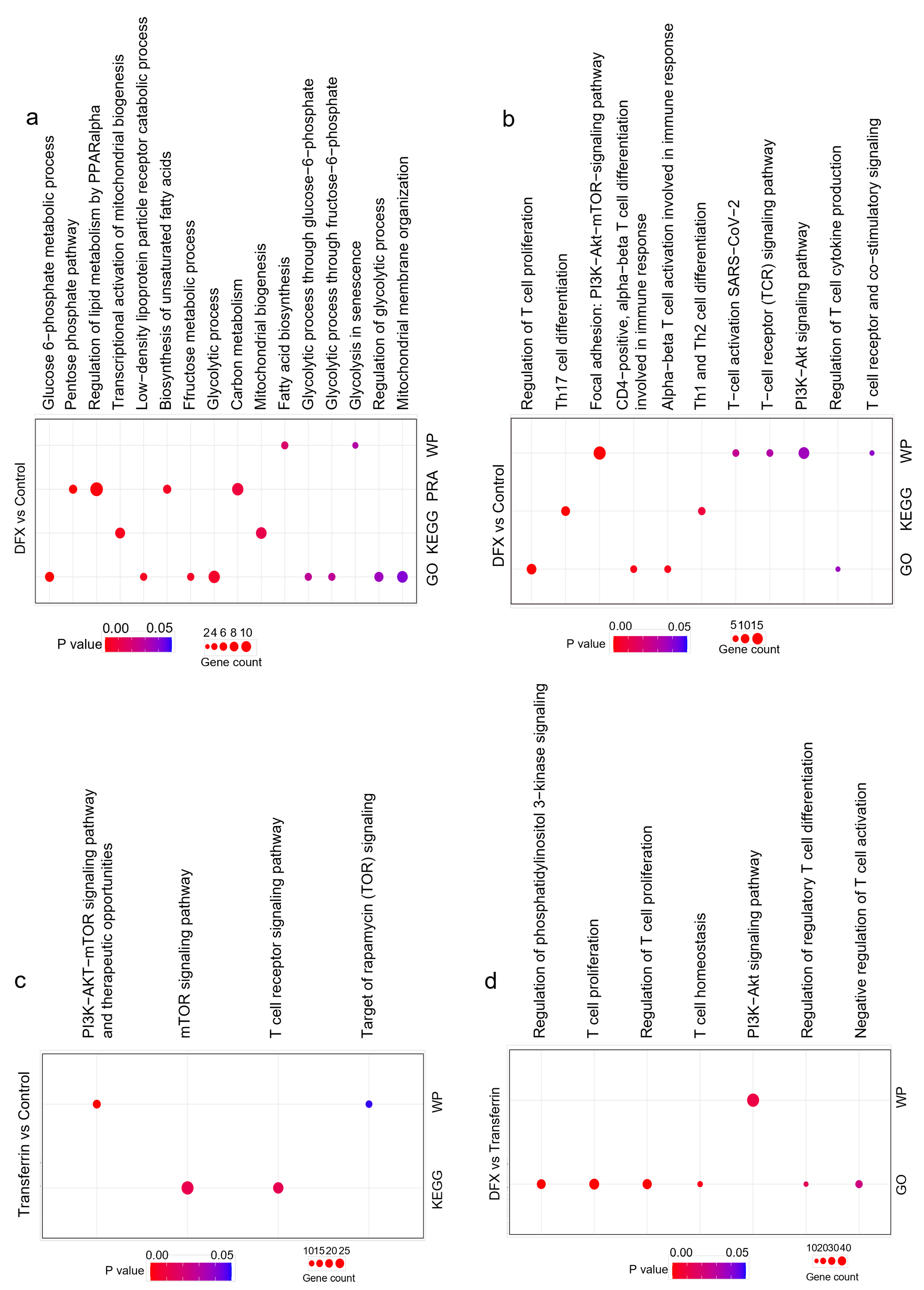

Supplement: Supplementary file 1 — Supporting Information [file CTM2-12-e999-s001.zip › Figure_1_supplnfo.docx]

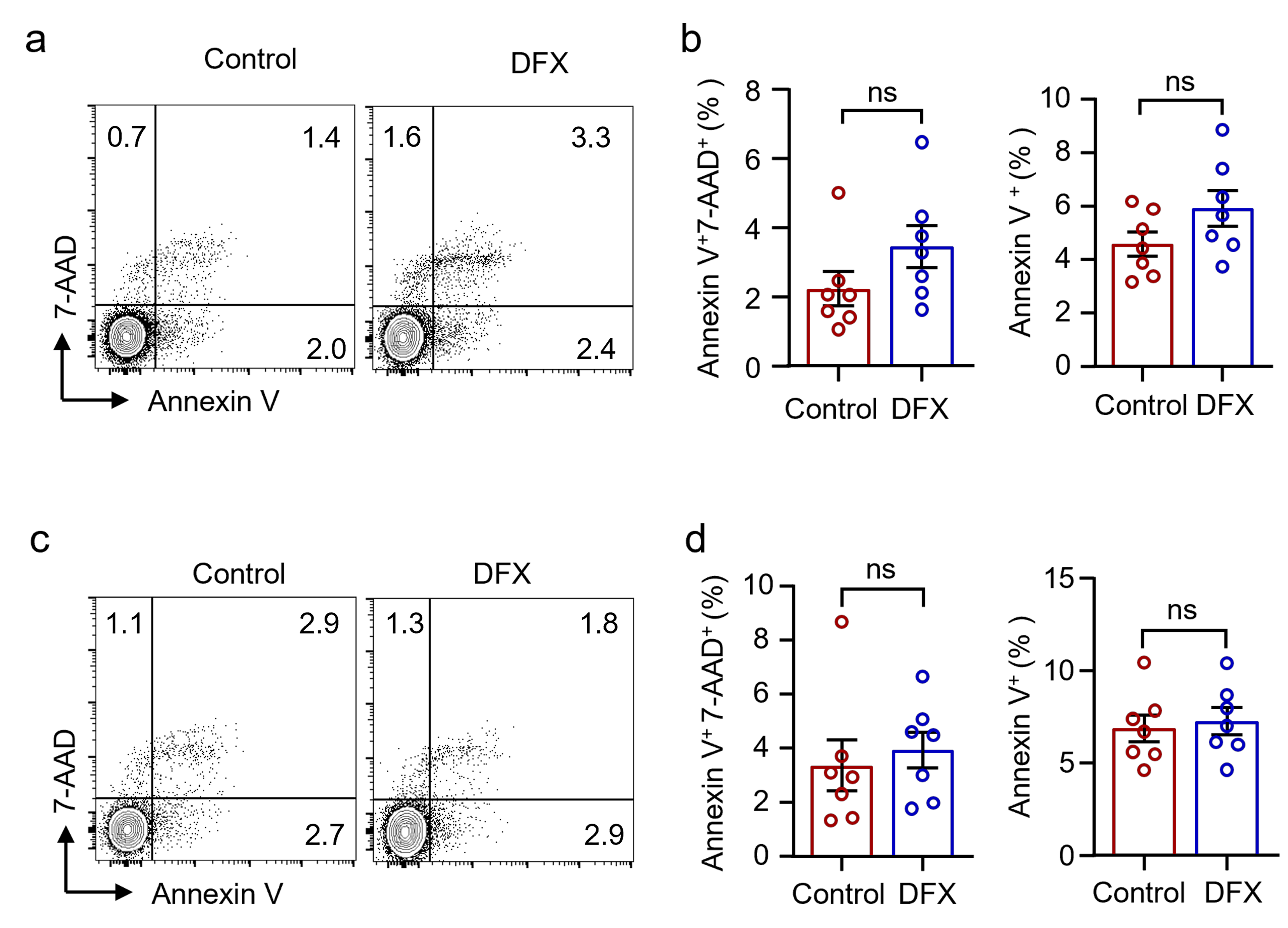

Supplement: Supplementary file 1 — Supporting Information [file CTM2-12-e999-s001.zip › Figure_2_supplnfo.docx]

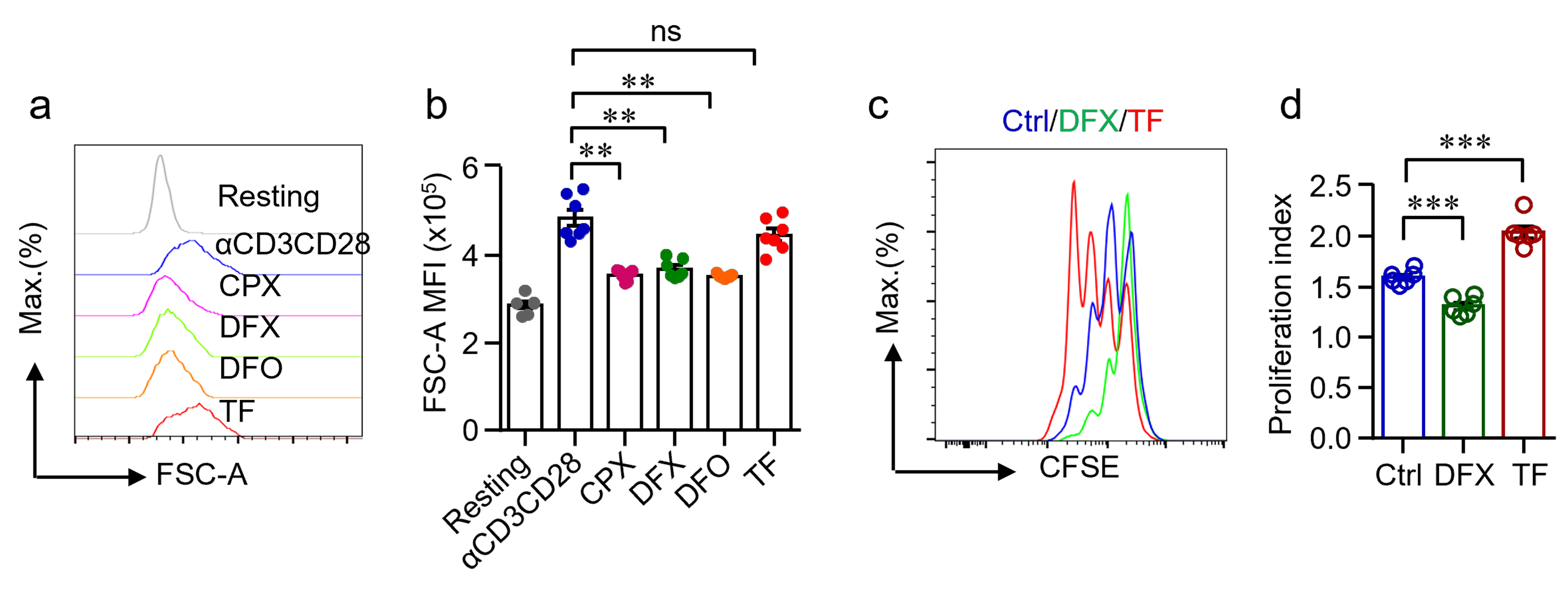

Supplement: Supplementary file 1 — Supporting Information [file CTM2-12-e999-s001.zip › Figure_3_supplnfo.docx]

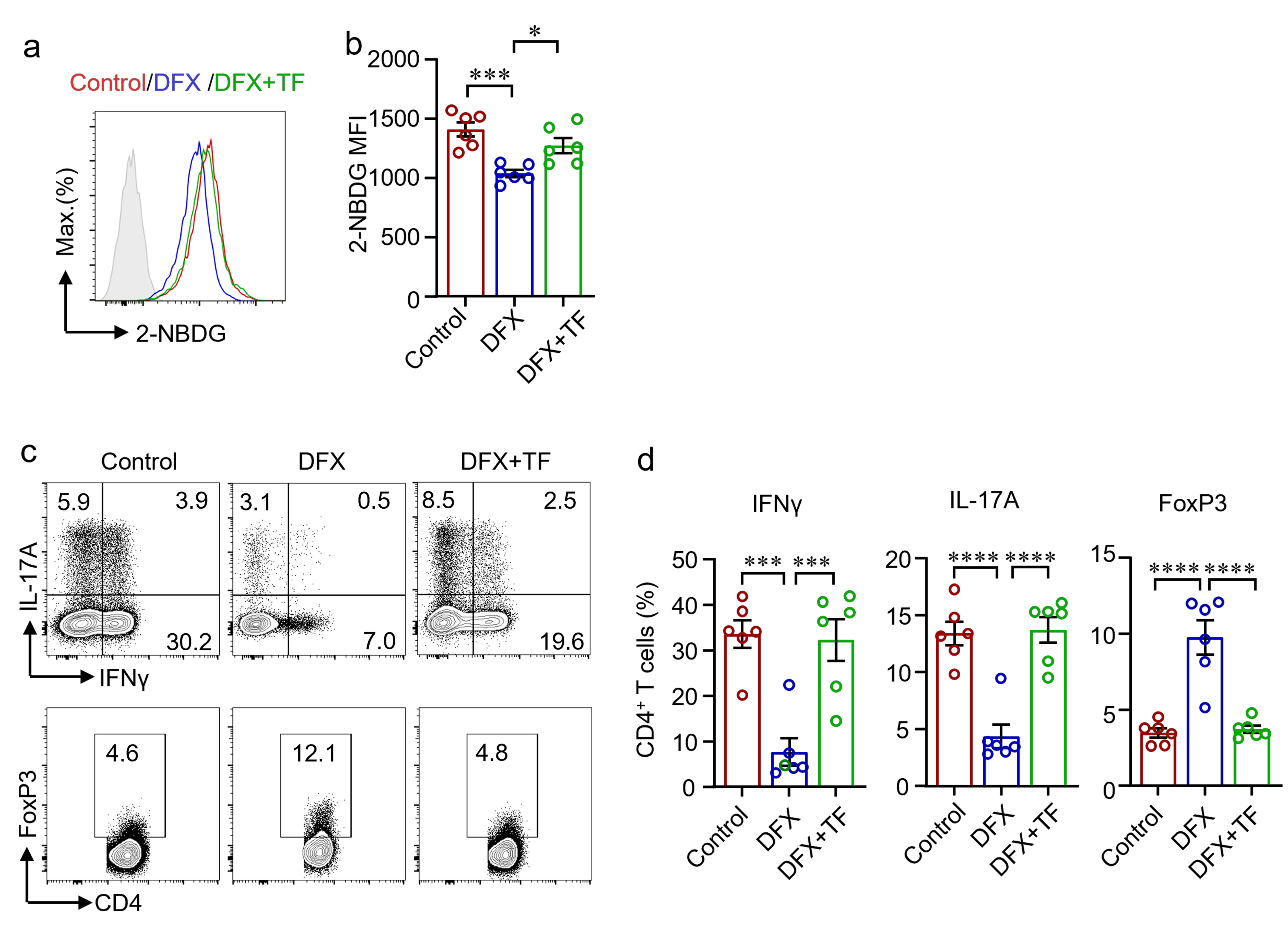

Supplement: Supplementary file 1 — Supporting Information [file CTM2-12-e999-s001.zip › Figure_4_supplnfo.docx]

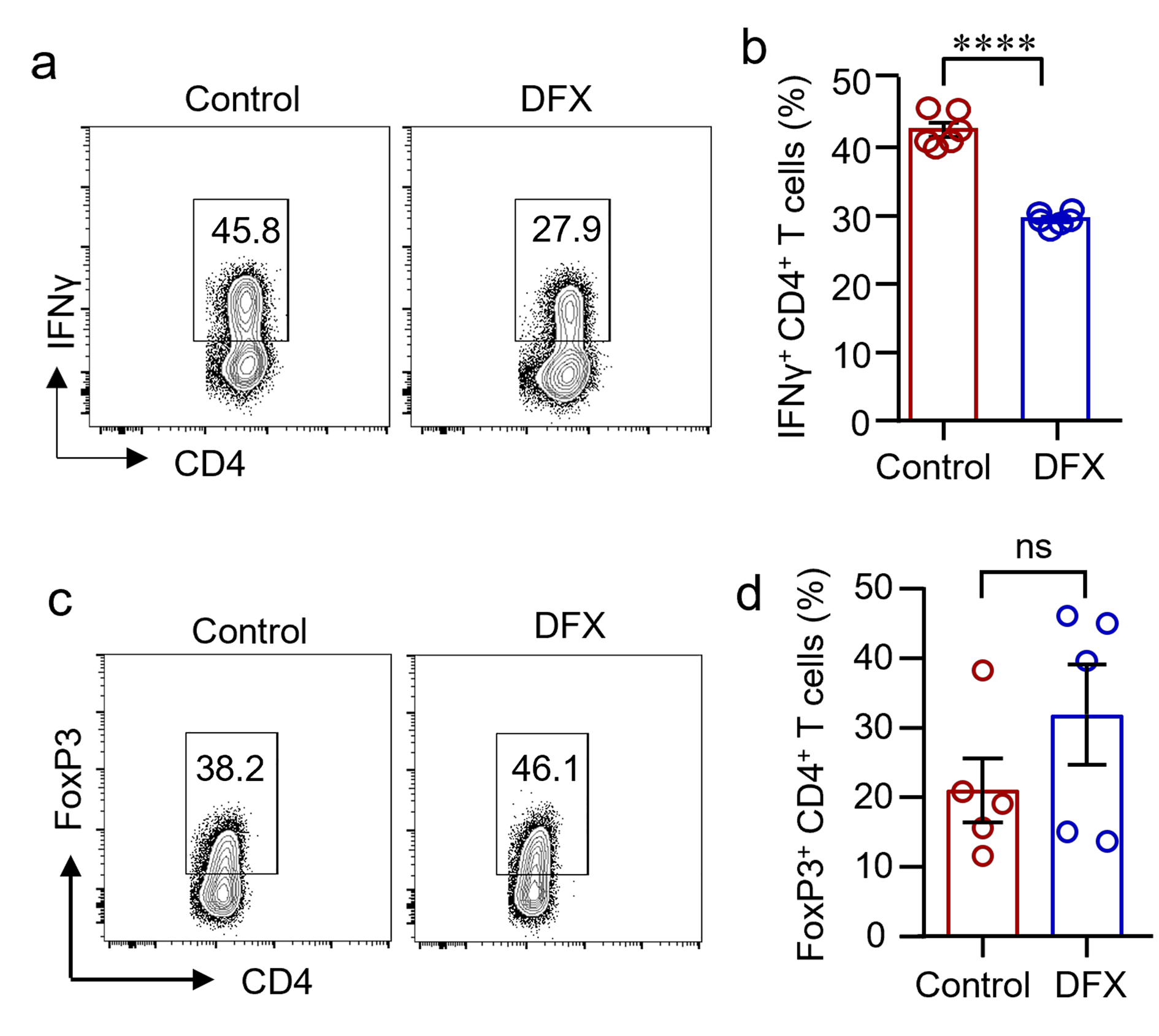

Supplement: Supplementary file 1 — Supporting Information [file CTM2-12-e999-s001.zip › Figure_5_supplnfo.docx]

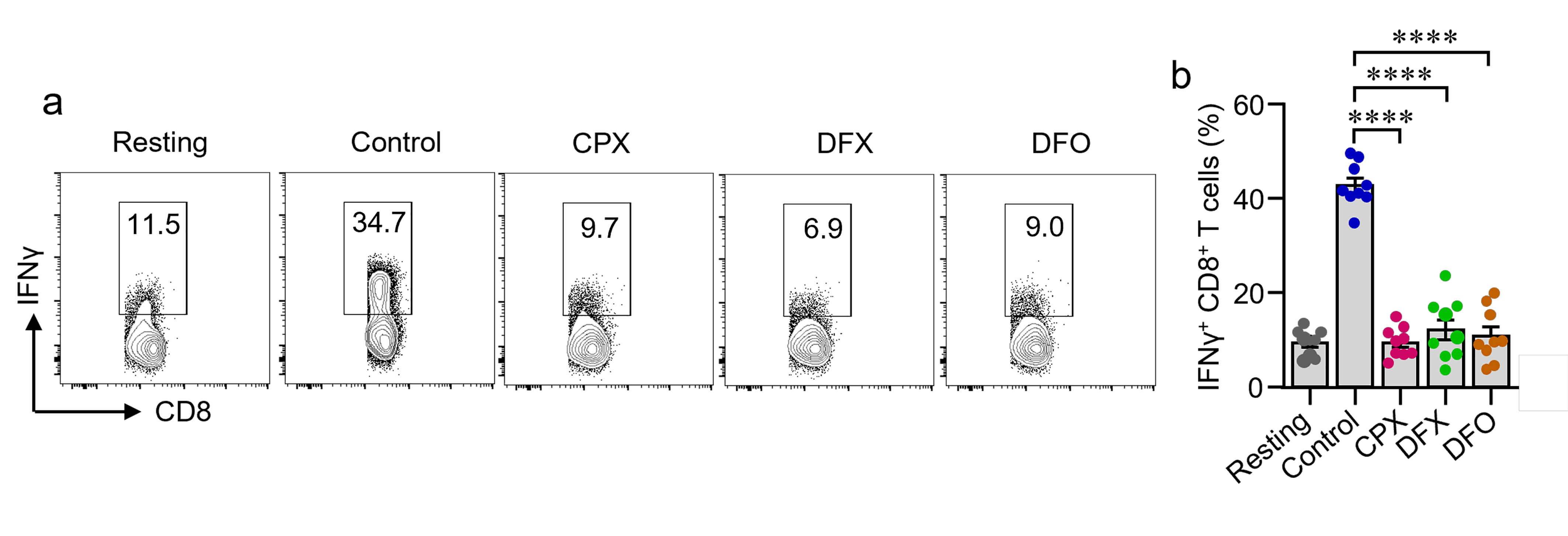

Supplement: Supplementary file 1 — Supporting Information [file CTM2-12-e999-s001.zip › Figure_6_supplnfo.docx]

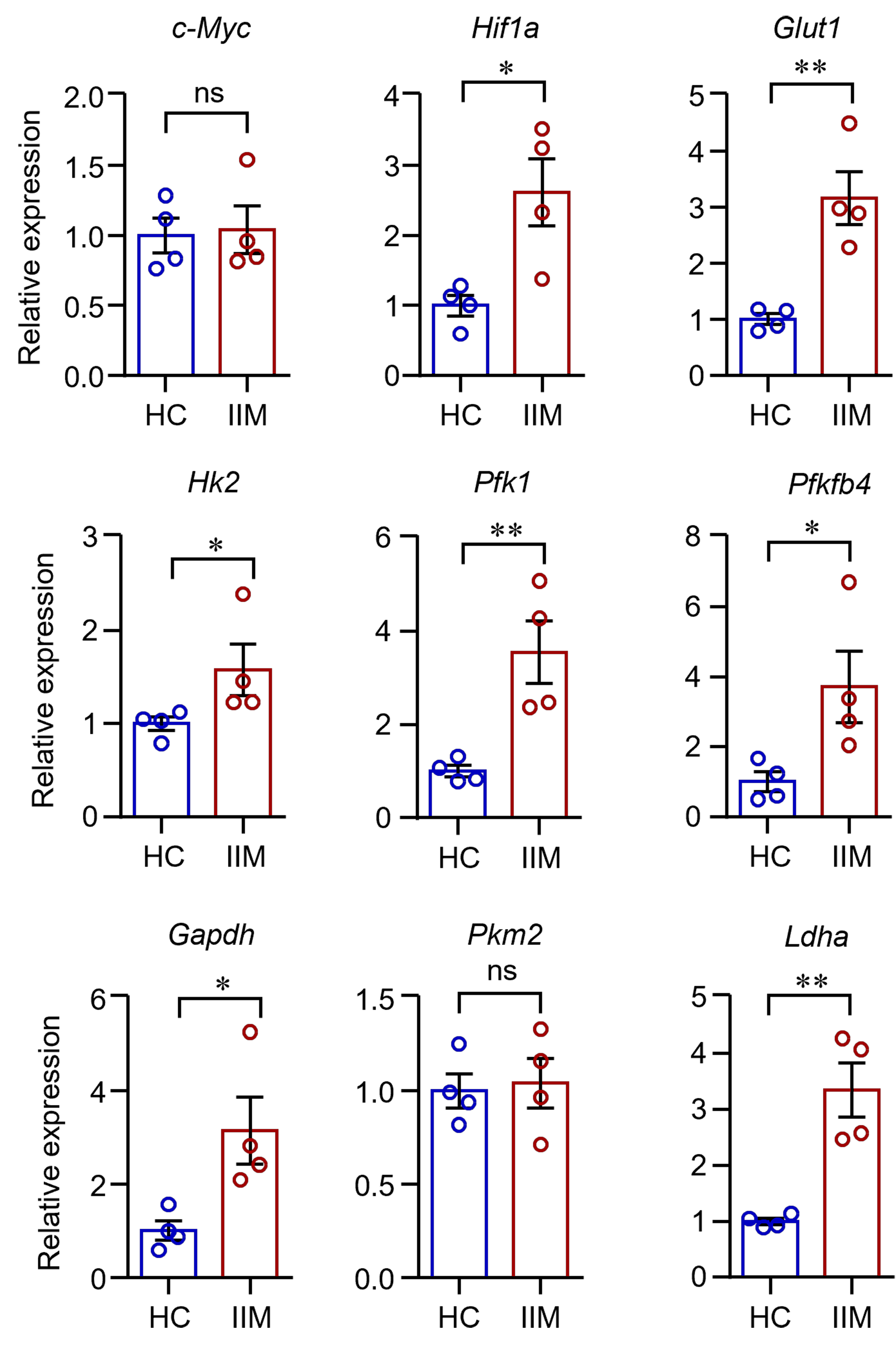

Supplement: Supplementary file 1 — Supporting Information [file CTM2-12-e999-s001.zip › Figure_7_supplnfo.docx]

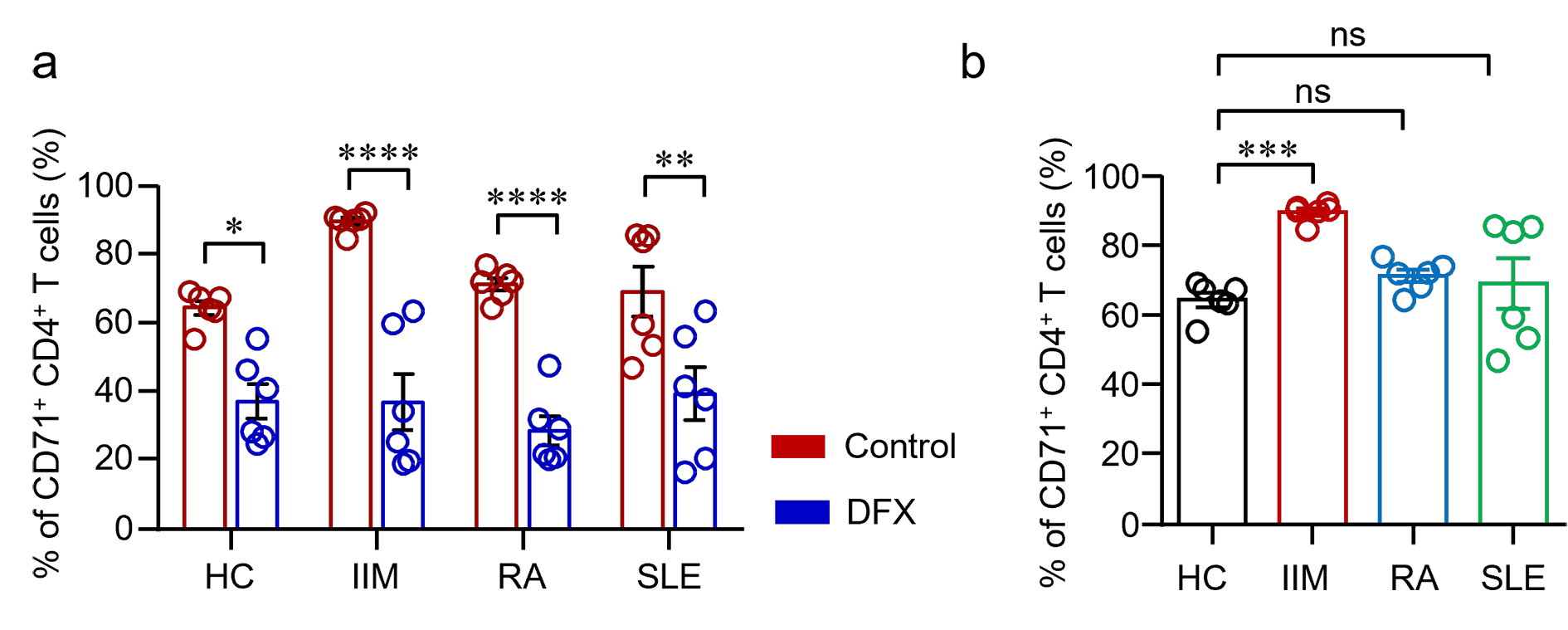

Supplement: Supplementary file 1 — Supporting Information [file CTM2-12-e999-s001.zip › Figure_8_supplnfo.docx]

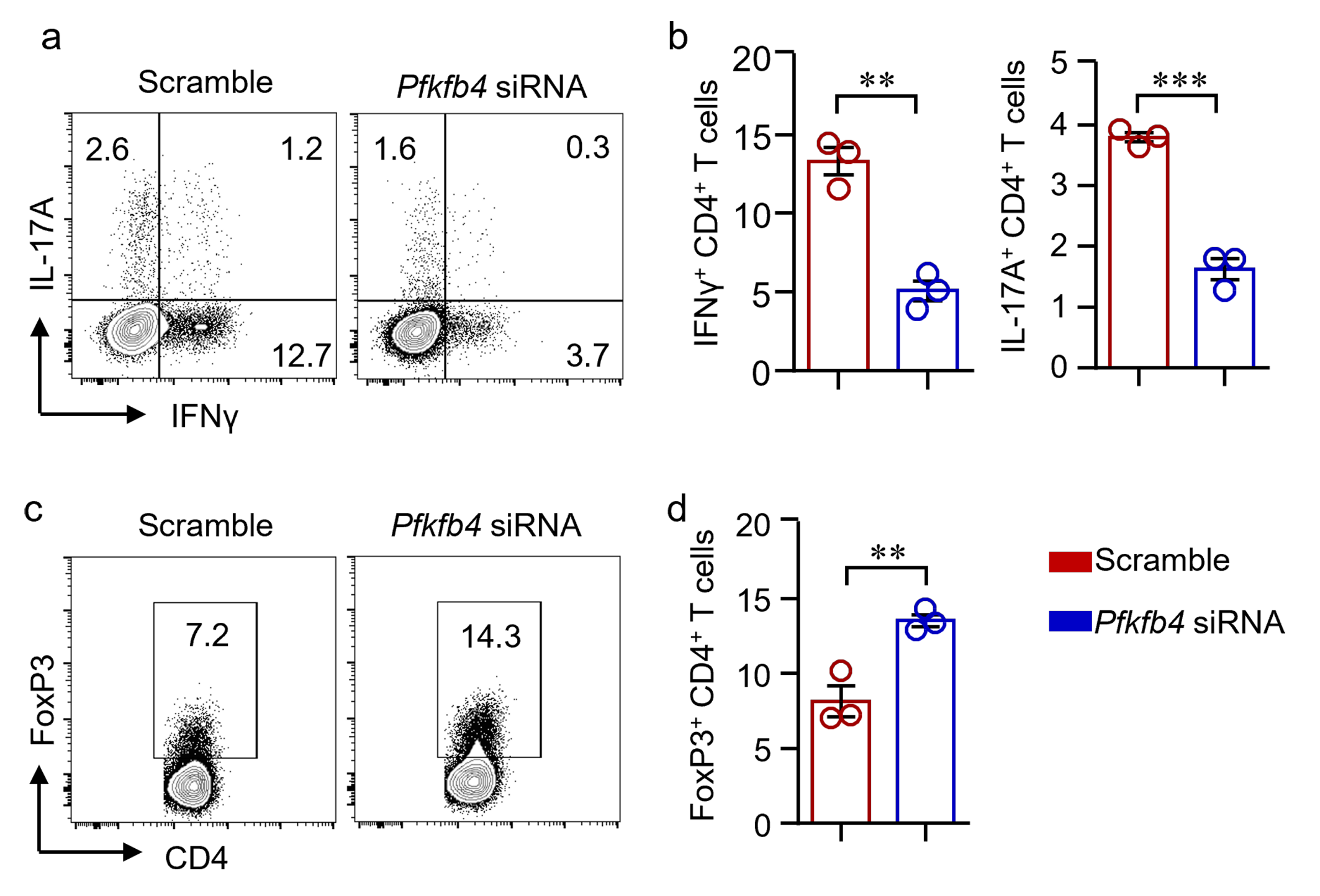

Supplement: Supplementary file 1 — Supporting Information [file CTM2-12-e999-s001.zip › Figure_9_supplnfo.docx]
